# NXPH4 mediated by m^5^C contributes to the malignant characteristics of colorectal cancer via inhibiting HIF1A degradation

**DOI:** 10.1186/s11658-024-00630-5

**Published:** 2024-08-20

**Authors:** Lei Yang, Jiawen Shi, Mingyang Zhong, Pingping Sun, Xiaojing Zhang, Zhengyi Lian, Hang Yin, Lijun Xu, Guyin He, Haiyan Xu, Han Wu, Ziheng Wang, Kai Miao, Jianfei Huang

**Affiliations:** 1 Clinical and Translational Research Center, Affiliated Hospital of Nantong University, Department of Oncology, Medical School of Nantong University, Nantong, 226001 Jiangsu China; 2 Department of Clinical Biobank and Institute of Oncology, Affiliated Hospital of Nantong University, Nantong, 226001 Jiangsu China; 3 Department of General Surgery, Affiliated Hospital of Nantong University, Nantong, 226001 China; 4 MOE Frontier Science Centre for Precision Oncology, University of Macau, Macau SAR, 999078 China

**Keywords:** NXPH4, m^5^C modification, Colorectal cancer (CRC), RNautophagy, HIF signaling pathway

## Abstract

**Objective:**

Colorectal cancer (CRC) is a form of malignancy that exhibits a comparatively elevated occurrence and fatality rate. Given the relatively slower progress in diagnostic and therapeutic approaches for CRC, there is a need to investigate more accurate and efficient biomarkers.

**Methods:**

Core regulatory genes were screened using the TCGA database, and the expression of neurexophilin 4 (NXPH4) and its prognostic implications were validated using tissue microarray staining. The assessment of NXPH4 functions involved a range of experiments, including cellular, organoid, and murine models. Furthermore, a regulatory network between m^5^C, NXPH4, and HIF1A was established through several in vitro experiments.

**Results:**

The overexpression of NXPH4 is associated with unfavorable prognoses in patients with CRC and hepatocellular carcinoma. Additionally, it facilitates the progression of malignant tumors both in laboratory settings and in living organisms of colorectal carcinoma. Our research also reveals that *NXPH4* mRNA can avoid degradation through RNautophagy, relying on an m^5^C-dependent mechanism. Moreover, NXPH4 amplifies the HIF signaling pathway and stabilizes HIF1A by competitively binding to PHD4.

**Conclusions:**

NXPH4, regulated by m^5^C, promotes malignant tumor progression and regulates the HIF pathway. Consequently, targeting NXPH4 through molecular therapies could potentially serve as an efficacious therapeutic strategy for the management of CRC exhibiting elevated NXPH4 expression.

**Graphical abstract:**

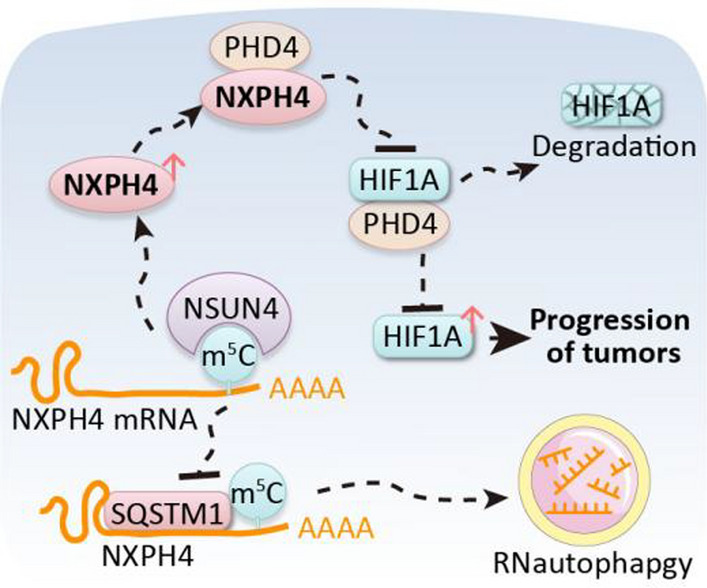

**Supplementary Information:**

The online version contains supplementary material available at 10.1186/s11658-024-00630-5.

## Introduction

Colorectal cancer (CRC) is a malignant tumor that arises within the colon or rectum, presenting with initial symptoms that are often subtle and easily dismissed [1, 2]. Despite advancements in therapy that provide limited alleviation for patients, the occurrence of CRC continues to be a prominent contributor to cancer-related mortality, accounting for approximately 1.9 million deaths annually worldwide [3]. The escalating prevalence of CRC emphasizes the critical need for more effective treatment strategies. In spite of advances in disease prevention, diagnosis, and treatment, the prognosis for CRC remains unfavorable due to late-stage diagnosis, recurrence, and drug resistance [4]. Therefore, discovering novel molecular targets for CRC might permit the development of more effective drugs.

Autophagy, a conserved cellular process, is crucial for preserving cellular homeostasis through the degradation and recycling of impaired cellular components [5], misfolded proteins [6], and intracellular pathogens [7]. Nevertheless, excessive activation or dysfunction of autophagy can intricately contribute to the initiation and progression of tumors [8]. Aberrant autophagy can facilitate tumor growth, thereby allowing increased tumor invasiveness and drug resistance [9]. While autophagy operates as a tumor suppressor by clearing damaged cellular constituents and constraining tumor growth, it can also support tumor survival and progression by providing nutrients and energy during metabolic stress or serving as a defense mechanism against therapeutic interventions [10]. A recently identified subtype of autophagy, known as RNautophagy, is responsible for the degradation of RNA within autophagosomes [11]. However, despite sharing regulatory proteins with microautophagy, their impact on tumor regulation remains poorly understood.

Neurexophilin 4 (NXPH4), a member of the neurexophilin family, displays widespread expression within the central nervous system and holds a critical role in regulating the formation and functionality [12, 13]. It can actively modulate synaptic plasticity and neurotransmitter release while regulating interneuronal signal transmission [12]. Moreover, NXPH4 plays a pivotal role in neurodevelopment and significantly contributes to the pathogenesis of neurodegenerative diseases [14]. Recent investigations have unveiled a multifaceted association between NXPH4 and various cancers, including lung cancer [15], kidney cancer [16], and bladder cancer [17]. Nevertheless, the relationship between NXPH4 and other digestive system carcinomas (DSCs) remains inadequately explored.

In this study, our results revealed elevated expression of NXPH4 in various DSCs, which correlated with unfavorable prognoses. Further experiments indicated that NXPH4 promotes the proliferation, migration, and invasion of CRC and hepatocellular carcinoma (HCC). Mechanistic investigations indicated *NXPH4* mRNA stability might be influenced by RNA modification and RNautophagy. Additionally, we found that NXPH4 protein competitively binds to PHD4, inhibiting HIF1A degradation. Collectively, these results underscore a new regulatory pathway in which NXPH4 plays a pivotal role in driving CRC and HCC progression.

## Materials and methods

### Cell culture

Cell lines (HEK293T *cat.CRL-3216*, NCM460 *cat. CP-H04.*, HCT116 *cat. CCL-247*, LoVo *cat CCL-229.*, SW480 *cat. CCL-228*, DLD-1 *cat. CCL‐221*, SW1116 *cat. CCL-233*, HCT15 *cat. CL-0097*, SW620 *cat. CCL-227*, LO-2 *cat. CL-0111*, HepG2 *cat. HB-8065*, Hep3B *cat. HB-8064*, SNU-182 *cat. CRL-2235*, and SK-Hep-1 *cat. HTB-52*) (Type Culture Collection of the Chinese Academy of Sciences, Shanghai, China) were cultivated in complete growth media, comprising 89% suitable culture medium (Gibco, Grand Island, NY), 10% fetal bovine serum (FBS) (Shuangru, Jiangsu, China) and 1% penicillin–streptomycin (NCM Biotech, Jiangsu, China) at 37 °C in a humidified incubator with 5% CO_2_.

### Data collection and bioinformatics analysis

RNA sequencing data from the TCGA and GEO databases and clinical data from the TCGA database (https://portal.gdc.cancer.gov/) were used. Survival assessments, encompassing overall survival (OS), disease-specific survival (DSS), and progression-free survival (PFS), were executed employing the Kaplan–Meier (KM) approach. KM curves were plotted using a log-rank *P* < 0.05. The “DESeq2” R software package [18] was employed for detecting differential gene expression, employing a > twofold change threshold and an adjusted *P*-value of < 0.05. Additionally, diagnostic performance validation of NXPH4 was undertaken using constructed receiver operating characteristic (ROC) curves. The area under the curve (AUC) for each gene was computed through the “pROC” R software package [19]. The prognostic relevance of NXPH4 in DSCs was ascertained. Differential expression genes (DEGs) were ranked in a prespecified list based on their correlation with NXPH4 by employing the “ggplot2” package [20] and Spearman’s rank correlation test. The R software package GSVA was utilized for analysis, with the parameter set to method = ‘ssgsea’. Spearman correlation was employed to assess the relationship between genes and pathway scores. Moreover, *NXPH4* expression from various tumors and adjacent samples from the TCGA dataset was investigated using the Xiantao Academy tool (https://www.xiantaozi.love). The relative *NXPH4* mRNA expression levels between the tumor and adjacent tissues were compared using the GEO datasets (GSE37182 [21] and GSE83889 [22], http://ncbi.nlm.nih.gov/geo/).

### Tissue samples and multiplex immunohistochemical staining (mIHC)

Patient tissue samples were retrieved from the Department of Clinical Biobank at Affiliated Hospital of Nantong University. All patients provided signed consent, and this study received approval from the Medical Ethics Committee of the Affiliated Hospital of Nantong University (no. 2023-l043; data 01/03/2023). The basic information of patients is listed in Table S1. Tissue sections were fixed in formalin and embedded in parafin and underwent deparaffinization and rehydration. Tissue microarray (TMA) was built utilizing individual paraffin-embedded tissues, following previously established protocols [23]. AR6 buffer (AR600, Akoya Biosciences, Marlborough, MA, USA) was employed for antigen retrieval. Subsequently, the TMA sections were subjected to overnight incubation at 4 °C using several primary antibodies. After thorough washing, the sections were exposed to secondary antibodies with distinct fluorophores. Comprehensive washing was performed between each staining stage. Finally, DAPI (Sigma-Aldrich, Saint Louis, MO) was utilized for counter-staining. The immunofluorescent signals were observed and documented employing the Vectra Automated Quantitative Pathology Imaging System (version 3.0, PerkinElmer, Waltham, MA, USA).

Staining intensity (score 0–100) was computed by inForm Image Analysis Software (v2.6.0, PerkinElmer). NXPH4 threshold levels were determined with X-tile (version: 3.6.1, Yale University) based on patients’ 5-year survival durations. Scores ranging from 0 to 29.5 were assigned to the category of low NXPH4 expression, while scores within the range of 29.6–100 were designated as high NXPH4 expression. Comprehensive information concerning antibodies, including dilution ratios and application details, can be found in Table S2.

### Patient-derived organoid culture

CRC Organoid Kit (Biogenous, Jiangsu, China) was applied to establish and maintain the CRC organoid. After dissecting the CRC tissues into small fragments of 1–3 mm^3^, they were agitated and digested for 90 min with Tumor Tissue Digestion Solution (Biogenous). After terminating the digestion process and lysing red blood cells, the cell pellets were washed thrice, then resuspended into Organoid Culture ECM (Biogenous) and seeded into plates. After the gel solidified, an organoid culture medium was added, and once the organoids reached a size visible to the naked eye, subculturing could be performed.

The ECM was disrupted using a pipette, and the organoids were subsequently collected by centrifugation. Following dissociation of the cells using Organoid Dissociation Solution (Biogenous), the organoids were resuspended back into the ECM. Lentiviral transduction could also be executed concurrently during subculturing. Two patient-derived organoids were analyzed in this study.

### RNA isolation and quantitative real-time PCR (qPCR) assays

Total RNA extraction was carried out with the RNA isolater Total RNA Extraction Reagent (Vazyme Biotech, Jiangsu, China) as per the provided manufacturer's guidelines. The extracted RNA was subjected to reverse transcription to generate cDNA, which was subsequently quantified through qPCR. Data acquisition was performed utilizing ChamQ Universal SYBR qPCR Master Mix (Vazyme). To ensure consistent loading, 18s rRNA was utilized as the internal control. The specific primer sequences for the targeted RNAs are detailed in Table S3.

### Chromatin immunoprecipitation (ChIP) assay

The ChIP assays were executed by adhering to protocols outlined in the ChIP Kit (Bersin Biotechnology, Guangzhou, China) to assess the binding interaction between HIF1A and the promoter region of potential target genes. Briefly, the cells were subjected to formaldehyde cross-linking, followed by isolation and sonication of chromatin to yield fragments spanning 200–500 bp. Subsequently, the processed chromatin was subjected to overnight precipitation with either HIF1A antibody or control IgG. After thorough washing and subsequent reverse cross-linking, DNA was purified and subjected to qPCR analysis to identify the specific genomic regions bound by HIF1A. Table S4 shows the primer sets utilized to target the regions encompassing potential HIF1A binding sites within the promoter.

### Dual-luciferase reporter assay

Transfection of HEK293T cells or the specified cells was performed with designated luciferase reporter plasmids and corresponding relevant plasmids. The Vazyme Dual-Luciferase Reporter Assay Kit was used to quantify firefly and Renilla luciferase activities after a 48-h incubation period. In order to normalize Renilla luciferase activity with firefly luciferase activity, a comparison was made between the two.

### Co-immunoprecipitation (co-IP) assay

Co-IP assays were conducted using the Co-IP Kit (Bersin). The specified cells underwent lysis using the lysis buffer supplemented with protease inhibitors. Upon removing cellular debris through centrifugation, the resulting supernatant was subjected to an overnight incubation at 4 °C alongside beads conjugated to anti-HIF1A/PHD4/NXPH4/IgG antibodies for subsequent immunoprecipitation. Following this, the complexes underwent thorough washing, elution, purification, and subsequent analysis through western blot.

### Western blot

Following various treatments, cellular total proteins were isolated. The total cellular proteins were extracted from the cells utilizing a lysis buffer supplemented with a protease inhibitor (Epizyme Biotech, Shanghai, China). Post-centrifugation, proteins in the soluble fractions were mixed with a loading buffer and denaturation at 95 °C for 10 min. Equivalent quantities of protein were loaded onto a polyacrylamide gel (Epizyme) for electrophoretic separation. Subsequently, they were transferred onto a PVDF membrane (Millipore, Darmstadt, Germany) and subjected to blocking with Protein Free Rapid Blocking Buffer (Epizyme) for 5 min. The blot was exposed to an overnight incubation at 4 °C with the primary antibody. After thorough washing, the membrane underwent incubation with an horseradish peroxidase (HRP)-conjugated secondary antibody for 1 h. Lastly, the blots were assessed using an ECL detection system (Epizyme) and quantified using the Image J software (National Institutes of Health). ACTB was selected as the internal control.

### RNA immunoprecipitation (RIP) assay and MS2-RIP assay

RIP assays were conducted using the RIP Kit (Bersin). The specified cells underwent lysis using the lysis buffer supplemented with protease and RNase inhibitors. Upon removing cellular debris through centrifugation, the resulting supernatant was subjected to an overnight incubation at 4 °C alongside beads conjugated to anti-m^5^C/NSUN4/SQSTM1/IgG antibodies for subsequent immunoprecipitation. Following this, the complexes underwent thorough washing, elution, purification, and subsequent analysis through qPCR.

MS2-RIP assays were conducted by following the guidelines outlined in a RIP Kit (Bersin). The 12 × MS2 stem loop was introduced at the 3′-untranslated region (UTR) terminus of the *NXPH4* mRNA. The MS2 stem loop can be recognized and bound by the MS2-GFP protein. Through this method, we can easily enrich mRNA containing the 12× MS2 stem loop using GFP antibodies. In this context, the designated cells underwent co-transfecting using target RNA expression vectors fused with the MS2 stem-loop and the MS2-GFP vector. The following procedures are analogous to the ones mentioned above.

### CCK-8, 5-ethyny-2′-deoxyuridine (EdU) assay and clone formation

The 2–4 × 10^3^ cells were dispensed into a 96-well plate and treated with the CCK-8 kit (Vazyme). After being incubated for 1 h, absorbance was measured to quantify cell viability. EdU (Beyotime, Shanghai, China) was performed in accordance with the instructions provided by the manufacturer. Transfected cells were subsequently placed into six-well plates at a density of 1000 cells/well for 10–14 days to evaluate their clone-forming capabilities.

### Targeted RNA demethylation system

The construction of the targeted RNA demethylation system followed established protocols involving enzyme digestions, PCR, and subcloning, as outlined in prior investigation [24]. The complete sequence of TET1/2 was integrated with dCas13b. To target *NXPH4* mRNA, suitable genomic RNAs (gRNAs) were designed and listed in Table S5. After this, the co-transfection of the dCas13-TET1/2 fusion expression vector and the gRNAs were executed within the cells.

### Transwell assay

Tumor cell migration and invasion were assessed through a Transwell assay. Uncoated polycarbonate inserts from Millipore were employed for migration, whereas for simulating an invasive environment, VitroGel RGD from TheWell Bioscience, North Brunswick, NJ, was used. In the upper chamber, 1–5 × 10^4^ cells were cultured in an FBS-free medium. The lower compartment contained a culture medium. After incubation, cells that did not migrate on the upper membrane surface were removed while cells that moved to the lower chamber were fixed with 4% paraformaldehyde solution and stained with crystal violet solution, and subsequent quantification.

### Plasmid construction, short hairpin RNA (shRNA), lentivirus, and cell transfection

NXPH4 sequence was inserted into a pCDH vector and combined with 12 × MS2 for the MS2-RIP assay. ShRNA molecules were designed and synthesized by Tsingke Biotechnology (Jiangsu, China). After annealing and purification, the fragments were integrated into the pLV-shRNA vector. This vector was co-transfected into HEK293T cells along with pMD2.G and psPAX2 plasmids to create recombinant lentiviral particles. These particles were then employed for cell transduction and gene delivery. Following exposure to lentiviral particles, the target cells were assessed for successful transduction after an appropriate incubation period. Table S6 provides detailed descriptions of the specific sequence used.

### Animal experiment

Six-week-old BALB/c nude mice were used for the xenograft procedures and were maintained under specific pathogen-free conditions. The experiments were approved by the Animal Care and Use Committee of Nantong University and Jiangsu Province Animal Care Ethics Committee (no. P20230224-029; data 24/02/2023). The mice received subcutaneous injections of SW1116 cells that had been stably transfected with lentiviruses containing shNC/shNXPH4. Each injection involved the administration of 5 × 10^6^ cells in a 200 μL solution. After 30 days, they were humanely euthanized, and their corresponding tumor weight was determined.

### Statistical analysis

Pearson’s *χ*^2^ test was used to determine the association of NXPH4 protein expressions with clinicopathological features. Survival assessments were performed using Cox regression models corresponding KM curves. Continuous and categorical factors were assessed using Mann–Whitney *U* test and *χ*^2^ test, respectively, and comparison between values was performed using Student's *t*-test and analysis of variance (ANOVA). Analyses were conducted using SPSS v22.0 (IBM, Chicago, USA), and data were visualized using GraphPad Prism v8.0 (GraphPad Software, San Diego, CA, USA). Experiments were repeated three times, in triplicate each time. Comparisons were determined to be statistically significant for *P* < 0.05 (*P < 0.05, **P < 0.01 and ***P < 0.001). Values are expressed as mean ± standard deviation.

## Results

### NXPH4 was identified as a novel prognostic biomarker of CRC

**Fig. 1:**
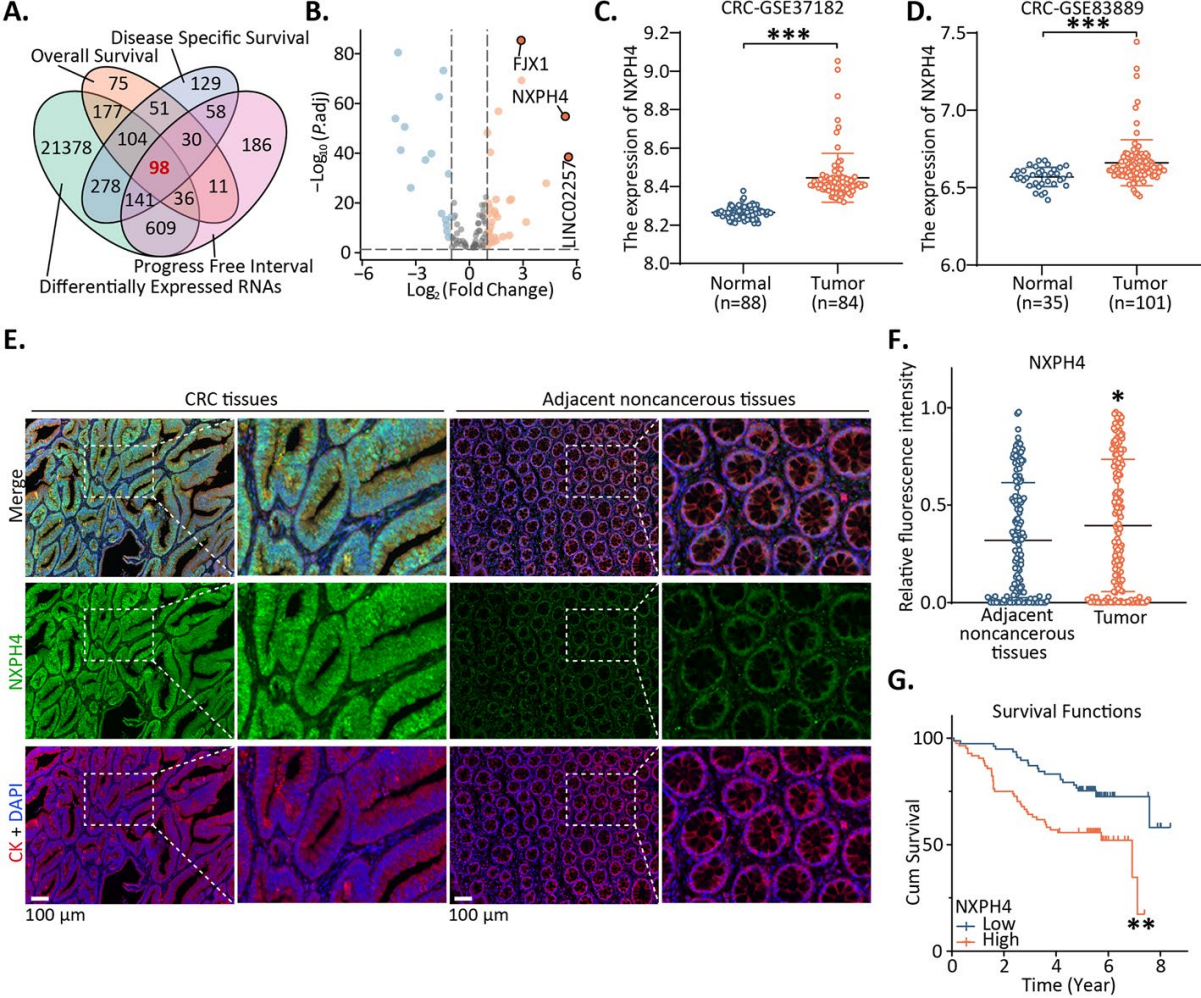
High *NXPH4* expression in CRC and HCC was associated with poor prognosis. **A**, **B** Analyses of candidate genes from the TCGA database by Venn diagrams and volcano plots. **C**, **D** Validation of *NXPH4* expression by GSE37182 and GSE83889 datasets. **E**,**F** The staining score analysis of* NXPH4* in CRC tissues is shown in comparison with paired adjacent noncancerous tissues. **G** Patients with high NXPH4 expression show significantly adverse 5-year survival than those with low NXPH4 expression. *P < 0.05, **P < 0.01, and ***P < 0.001

Overall survival (OS), disease specific survival (DSS) and progression-free interval (PFI) were performed to explore relevant genes in CRC patient samples. Target genes that were differentially expressed were discerned between CRC and peritumoral tissues, and these findings were visually represented using a four-way Venn diagram. Among the 98 genes displaying differential expression, further analysis using volcano plots indicated a notable upregulation of *NXPH4* (Fig. 1A, B). A pan-cancer analysis demonstrated the consistent upregulation of *NXPH4* across various DSCs, suggesting its diagnostic potential in cholangiocarcinoma, esophageal cancer, liver cancer, and gastric cancer (Fig. S1).

*NXPH4* overexpression was further determined by assessing its expression between the adjacent and cancer tissues using the GSE37182 and GSE83889 datasets from the GEO database (Fig. 1C, D). TCGA data analysis revealed elevated *NXPH4* expression in tumors with advanced T and N classifications, as well as higher tumor grade in both CRC and HCC (Fig. S2A-B), highlighting *NXPH4*’s strong correlation with unfavorable prognosis in CRC and its association with OS and DSS in HCC (Fig. S2C–H). We evaluated the expression of *NXPH4* in 21 pairs of CRC and HCC tissues, along with their adjacent noncancerous tissues, by qPCR. A total of 19 of 21 (90.5%) cancerous specimens had higher *NXPH4* mRNA levels than the adjacent noncancerous tissues in CRC group, and 17 of 21 (80.9%) in HCC group (Fig. S2I, J). NXPH4 was suggested to be promising diagnostic biological markers in this disease (Fig. S2K–R). To confirm this phenomenon at the protein level, immunofluorescence staining and analysis of our CRC cohort was performed. The significant upregulation of NXPH4 expression in tumor tissues was linked to poor prognosis (Fig. 1E–G). The NXPH4 protein is primarily concentrated in the cytoplasm, with a lesser amount also present in the cell membrane. The corresponding statistical results indicated NXPH4 might serve as an independent prognostic variable in CRC (Tables 1, 2).

**Table 1:** Relationship between the expression of NXPH4 and clinicopathological characteristics in CRC

Characteristic	*n*	Low or no expression	High expression	Pearson *χ*^2^	*P*
Total	161	77 (47.83)	84 (52.17)		
Age				0.054	0.817
< 60	55	27 (49.09)	28 (50.91)		
≥ 60	106	50 (47.17)	56 (52.83)		
Gender				0.181	0.67
Female	60	30 (50.00)	30 (50.00)		
Male	101	47 (46.53)	54 (53.47)		
T				8.044	0.045*
Tis and T1	6	5 (83.33)	1 (16.67)		
T2	38	22 (57.89)	16 (42.11)		
T3	114	50 (43.86)	64 (56.14)		
T4	3	0 (0.00)	3 (100.00)		
N				0.609	0.738
N0	104	51 (49.04)	53 (50.96)		
N1	46	20 (43.48)	26 (56.52)		
N2	11	6 (54.55)	5 (45.45)		
M				4.21	0.040*
M0	153	76 (49.67)	77 (50.33)		
M1a and M1b	8	1 (12.50)	7 (87.50)		
TNM stage				8.764	0.033*
0 and Ia and Ib	38	24 (63.16)	14 (36.84)		
IIa and IIb	63	26 (41.27)	37 (58.73)		
IIIa and IIIb and IIIc	52	26 (50.00)	26 (50.00)		
IV	8	1 (12.50)	7 (87.50)		
Differentiation				6.896	0.009**
Well and middle	135	69 (51.11)	66 (48.89)		
Poor	14	2 (14.29)	12 (85.71)		
Unknown	12				
Tumor site				0.321	0.571
Colon	111	50 (45.05)	61 (54.95)		
Rectum	46	23 (50.00)	23 (50.00)		
Unknown	4				

*P < 0.05, **P < 0.001

**Table 2:** Univariate and multivariate analyses of prognostic factors for overall survival in CRC

Variable	Univariate analysis				Multivariate analysis			
	HR	*P*	95% CI		HR	*P*	95% CI	
NXPH4 expression								
High versus low	2.361	0.002**	1.377	4.048	1.800	0.044*	1.017	3.186
Age (years)								
≤ 60 versus > 60	1.306	0.073	0.975	1.749				
Gender								
Male versus female	1.363	0.050	1.001	1.857				
T								
Tis-T1–T2 versus T3–T4	1.484	< 0.001***	1.226	1.795	3.152	0.009**	1.327	7.490
N								
N0 versus N1–N2	1.583	< 0.001***	1.335	1.878				
M	8.137	< 0.001***	3.721	17.793	5.877	< 0.001***	2.457	14.055
M0 versus M1								
TNM stage								
0–I–II versus III–IV	1.530	0.101	0.921	2.542				
Differentiation								
Well and middle versus poor	4.855	< 0.001***	2.482	9.497	2.900	0.003**	1.444	5.826
Tumor site								
Colon versus rectum	1.468	0.169	0.849	2.538				

^*^P < 0.05, **P < 0.01, ***P < 0.001

### NXPH4 accelerated aggressiveness of CRC and HCC

**Fig. 2:**
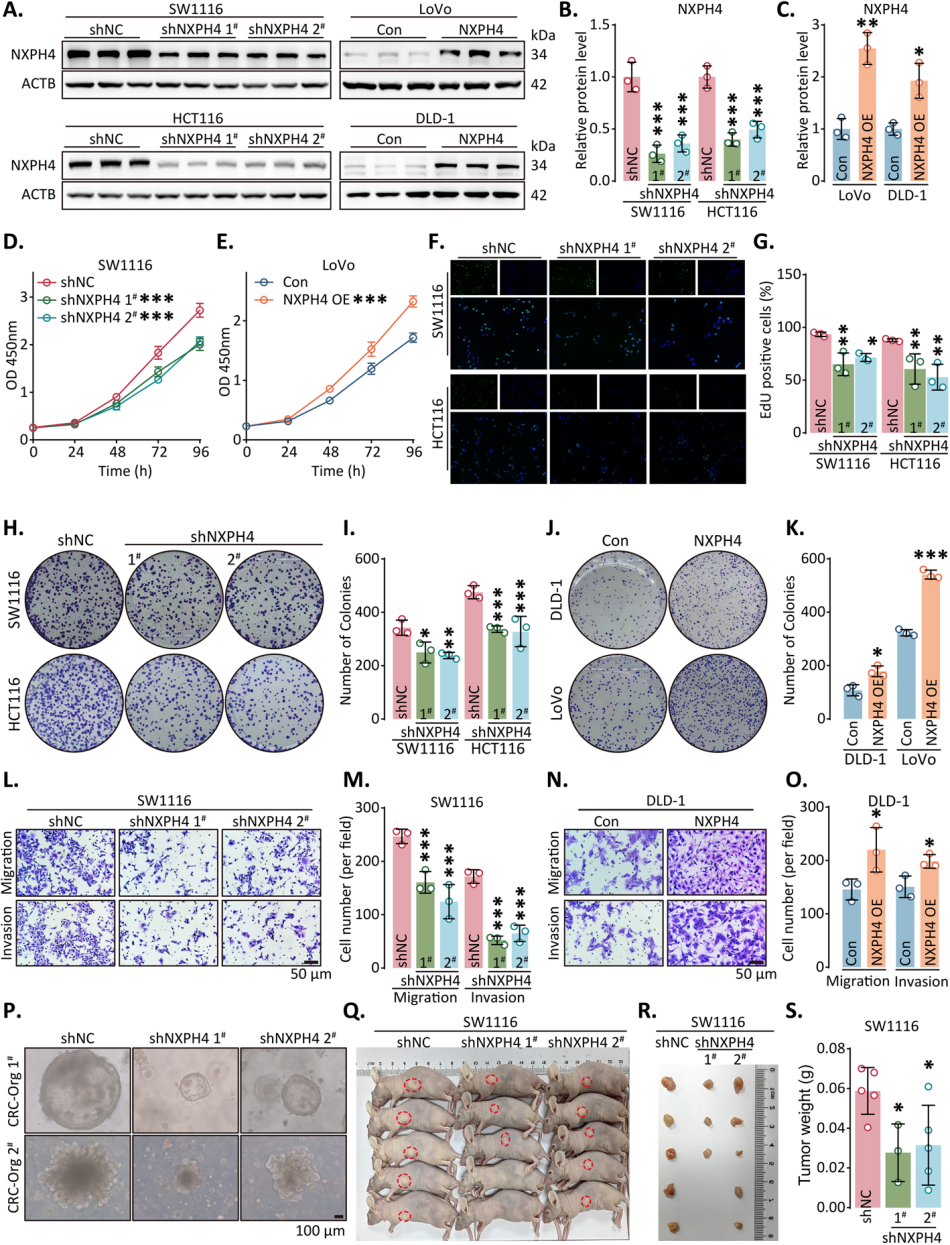
NXPH4 accelerated aggressiveness of CRC. **A**–**C** NXPH4 was knocked down in SW1116 and HCT116 cells and overexpressed in LoVo and DLD-1 cells using western blot analysis. **D**, **E** The proliferation of cells following the knockdown or overexpression of NXPH4 was assessed via CCK-8 assay. **F, G** Following the silence of NXPH4, the EdU assay measured DNA synthesis capacity. **H**–**K** The clonogenic assay evaluated clonal formation ability after overexpression or knockdown of NXPH4.** L**–**O** After NXPH4 is knocked down or overexpressed, the Transwell assay measures invasion and migration. **P** Here are representative pictures of CRC organoids after NXPH4 has been knocked down. **Q**, **R** Nude mice were injected subcutaneously with SW1116 cells and tumor formation monitored over a period of several weeks. **S** The tumor weight was measured as indicated. Data were presented as the mean ± standard deviation. *P < 0.05, **P < 0.01, and ***P < 0.001

To elucidate the oncogenic role of NXPH4, we devised and established shRNA, overexpression vectors, and their respective lentiviral systems. We assessed the protein levels of NXPH4 in CRC and HCC cell lines (Fig. S3A–C). Subsequently, cell lines displaying elevated NXPH4 levels (SW1116, HCT116, HepG2 and SK-Hep-1) were transfected with shRNAs, while cell lines exhibiting low NXPH4 expression (LoVo, DLD-1, Hep3B and SNU-182) were subjected to ectopic expression. Stable cell lines were generated through lentivirus-mediated approaches, facilitating the overexpression or knockdown of NXPH4. These stable cell lines were validated using qPCR and western blot analyses (Fig. 2A–C; Fig. S3D–G).

The outcomes of the CCK-8 assays highlighted notable reduction in cells’ proliferative capability with diminished NXPH4 levels. Conversely, overexpression of NXPH4 substantially bolstered the proliferation of CRC cells (Fig. 2D, E; Fig. S4A, B). Further support was provided by the EdU assay, which indicated a decrease in EdU-positive cells following NXPH4 reduction (Fig. 2F, G). On the other hand, ectopic expression of NXPH4 contributed to an accelerated rate of DNA replication in the investigated cancer cells (Fig. S4C, D). Furthermore, findings from the colony formation assay underscored that NXPH4 knockdown decreased the clonogenic capacity of tumor cells, whereas the counterpart assay revealed an increased number of colonies in cells overexpressing NXPH4 compared to the control group (Fig. 2H–K). Subsequent assessment using the Transwell assay revealed a substantial enhancement in CRC cell migration and invasion upon NXPH4 upregulation (Fig. 2L–O; Fig. S4E–H). Conversely, a reverse effect was observed upon NXPH4 downregulation.

To replicate the characteristics of the tumor microenvironment, particularly hypoxia, in vitro, we established a CRC organoid model. Lentivirus-transfected organoids showed significantly reduced growth and size in the NXPH4 knockdown group (Fig. 2P; Fig. S5). Conversely, overexpressing NXPH4 significantly enhanced organoid growth, and these findings were validated in vivo by implanting cells with stable NXPH4 downregulation subcutaneously, thereby confirming our earlier observation of reduced tumor cell proliferation upon NXPH4 silencing (Fig. 2Q–S). Similarly, we observed the same function of NXPH4 in HCC cell lines (Fig. S6). Collectively, our findings strongly support NXPH4 as a tumor-promoting gene in both CRC and HCC.

### ***NXPH4*** mRNA stability was maintained in m^5^C-dependent manner

**Fig. 3:**
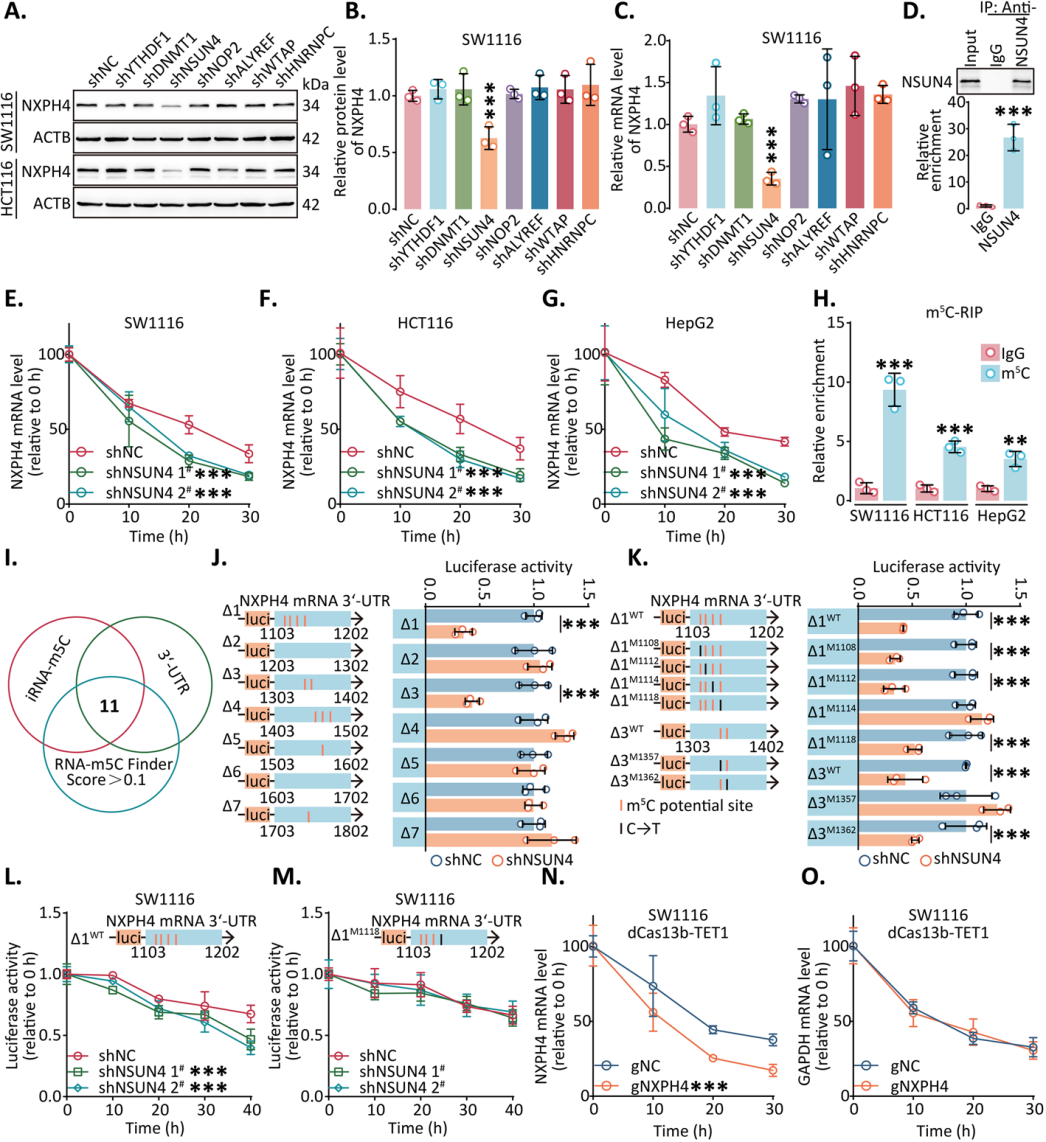
*NXPH4* mRNA stability was maintained by NSUN4 (**A**–**C**) SW1116 and HCT116 cells were transfected with indicated lentiviruses and the expression of NXPH4 was analyzed by western blot and qPCR. **D** The enrichment of *NXPH4* mRNA was assessed using the RIP assay and qPCR. IgG control was used to estimate the level of enrichment. **E**–**G** The stability of *NXPH4* mRNA in SW1116 and HCT116 cells transfected with NSUN4-shRNA was determined by qPCR after the initiation of RNA transcription was blocked with α-amanitin. **H** The enrichment of *NXPH4* mRNA was assessed using the MeRIP assay and qPCR. IgG control was used to estimate the level of enrichment. **I** Venn diagram of the potential m^5^C site by three databases.** J–M** Following treatment with indicated lentiviruses, luciferase activity of full-length and mutation NXPH4 was measured. **N–O** The stability of NXPH4 and GAPDH mRNAs following lentivirus transfection was examined by qPCR. Data were presented as the mean ± standard deviation. **P < 0.01, and ***P < 0.001

To investigate the potential mechanisms underlying the upregulation of NXPH4, we analyzed the correlation between its expression and various epigenetic-related genes using the TCGA database, which revealed 12 genes that exhibited significant correlations with NXPH4 expression in both CRC and HCC datasets (Fig. S7A). Further assessments were performed for determining these genes’ differential expressions in cancerous and non-cancerous tissues in the TCGA and CPTAC databases, revealing that YTHDF1, DNMT1, NSUN4, NOP2, ALYREF, WTAP and HNRNPC demonstrated notable upregulation in tumor tissues at both the protein and RNA levels and could be promising targets for further investigation (Fig. S7B–G). Subsequently, we transfected CRC cell lines with shRNAs and control lentiviruses to verify the influence of the potential regulators on *NXPH4* mRNA. Changes observed in protein and RNA levels indicated a direct regulatory role of NSUN4 on *NXPH4* mRNA, which were successfully validated in HCC cell lines (Fig. 3A–C; Fig. S8). Furthermore, the RIP assay revealed that NSUN4 antibody enrichment was associated with *NXPH4* mRNA, indicating the direct binding of NSUN4 to *NXPH4* mRNA (Fig. 3D; Fig. S9A). To delve deeper into the regulatory mechanism, we administered α-amanitin, an RNA synthesis inhibitor, to cells to observe the rate of RNA degradation. The results demonstrated a significant acceleration in the degradation of *NXPH4* mRNA upon NSUN4 silencing (Fig. 3E–G; Fig. S9B–D).

NSUN4 is an established RNA methyltransferase recognized for its role in catalyzing m^5^C modification on RNA molecules. To explore the presence of m^5^C modification on *NXPH4* mRNA, we conducted m^5^C-specific RIP experiments (Fig. 3H). Using iRNA-m^5^C and RNA-m^5^C Finder online tools, we identified potential modification sites within the 3′ untranslated region (UTR) of *NXPH4* mRNA, which yielded 11 potential modification sites, as indicated by the Venn diagram (Fig. 3I). Truncation plasmids were meticulously designed and employed in dual-luciferase assays to validate these putative modification sites, which confirmed the presence of m^5^C modification sites within the regions 1103–1202 and 1303–1402 of *NXPH4* mRNA (Fig. 3J). Dual-luciferase assays using these constructs revealed potential m^5^C modification sites at positions 1114 and 1357 (Fig. 3K). Additionally, dual-luciferase assays were used to quantify the decay rate of luciferase mRNA following NSUN4 downregulation, and no considerable alteration in the degradation rate was observed after mutation at positions 1114 or 1357 (Fig. 3L, M; Fig. S9E, F).

To confirm the protective effects of m^5^C on *NXPH4* mRNA against degradation, we used a pre-established targeted RNA demethylation system to counteract the overall effects caused by knockdown. TET1 and TET2 were used as demethylase enzymes, while dCas13b-TET1/2 was recruited to specific regions of *NXPH4* mRNA via a guide RNA (gNXPH4). This arrangement facilitated the action of demethylases at distinct sites. Consequently, co-transfection of dCas13b-TET1/TET2 and gRNA into cells led to a substantial acceleration in the decay rate of NXPH4, underscoring the suppressive impact of m^5^C modification on *NXPH4* mRNA degradation (Fig. 3N, O; Fig. S9G–P). To further understand m^5^C's biological role in *NXPH4* mRNA, we conducted CCK-8, colony formation and Transwell assays. The outcomes demonstrated that the removal of m^5^C modifications from native *NXPH4* mRNA severely impeded the capacity of tumor cells to proliferate, migrate, and invade (Fig. S10, 11).

**Fig. 4:**
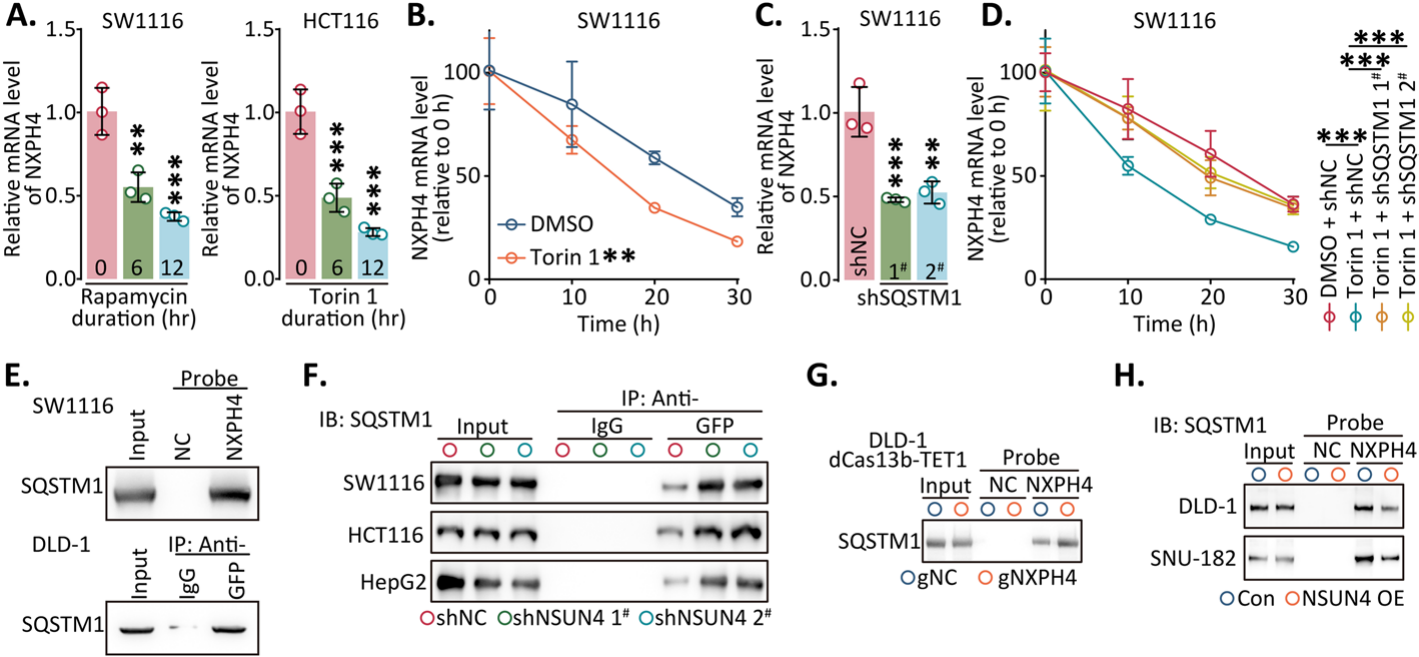
*NXPH4* mRNA escaped from RNautophagy in m^5^C-dependent manner.** A** The levels of *NXPH4* mRNA after treatment with Rapamycin and Torin 1 were determined using qPCR. **B** The stability of *NXPH4* mRNA after Torin1 treatment was assessed using qPCR. **C** The level of *NXPH4* mRNA expression after SQSTM1 downregulation was determined by qPCR. **D** Following Torin 1 and SQSTM1-shRNA treatment, *NXPH4* mRNA was assessed by qPCR. **E**–**H** The *NXPH4* mRNA-specific probe or anti-GFP antibody was used for RNA pulldown or GFP-RIP assay respectively, and the enrichment of SQSTM1 was detected by western blot. Data were presented as the mean ± standard deviation. **P < 0.01, and ***P < 0.001

Dysregulated autophagy has been implicated as a contributor to cancer progression[25]. Several RNAs, including SQSTM1 [26] and LAMP2C [27], can be subjected to degradation through autophagy-dependent pathways (RNautophagy). Activation of autophagy with rapamycin or Torin 1 resulted in a notable reduction in *NXPH4* mRNA levels, which was significantly reversed upon autophagy inhibition by chloroquine or bafilomycin A1 (Fig. 4A; Fig. S12A, B). Autophagy activation led to a significant decrease in the stability of *NXPH4* mRNA, notably shortening its half-life (Fig. 4B; Fig. S12C–I), which suggested that autophagy could regulate the expression of NXPH4.

Among the potential mediators examined, the study focused on six common mediators, and the results strongly highlighted the prominent role of SQSTM1 in *NXPH4* mRNA degradation (Fig. 4C; Fig. S12J–L). The activation of autophagy indeed correlated with a downregulation of *NXPH4* mRNA expression, which could be rescued by inhibiting SQSTM1 (Fig. 4D; Fig. S12M–O). Silencing of SQSTM1 effectively counteracted the shortened half-life of *NXPH4* mRNA caused by Torin 1. Furthermore, a direct interaction between *NXPH4* mRNA and SQSTM1 was observed by RNA pulldown and MS2-RIP (Fig. 4E). This interaction was notably enhanced after the removal of m^5^C modification or knockdown of NSUN4 (Fig. 4F, G). Conversely, overexpression of NSUN4 notably attenuated the interaction between *NXPH4* mRNA and SQSTM1 (Fig. 4H). In summary, these findings provide evidence that the stability of *NXPH4* mRNA is influenced by m^5^C modification, potentially affecting the direct binding between *NXPH4* mRNA and SQSTM1.

### NXPH4 enhanced HIF signaling pathway

**Fig. 5:**
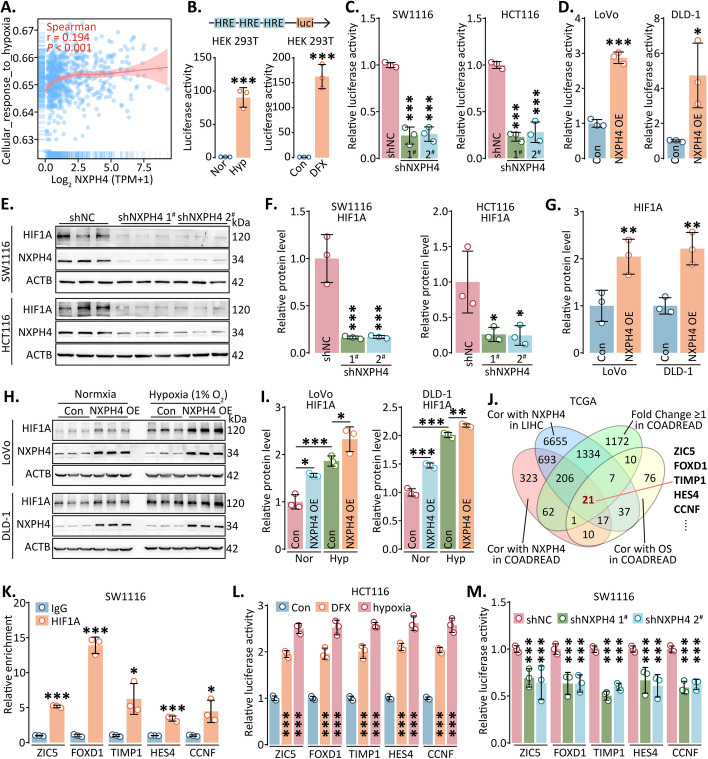
NXPH4 enhanced HIF signaling pathway.**A** Signaling pathway enrichment analysis of NXPH4. **B** Construction and confirmation of HIF reporter. **C**,**D** Luciferase activity of HIF reporter was measured after being treated with indicated lentivirus.** E–I** Following transfection with the indicated lentivirus or treatment, Western blot analysis of NXPH4 and HIF1A expressions was performed in CRC cells.**J**Venn diagram analyses of candidate genes related with NXPH4 from the TCGA database. **K** Potential target genes were found to be bound to HIF1A by ChIP and qPCR assays. **L, M** Target gene reporter activity was measured after treatment with indicated treatments. Hypoxia: 1% oxygen; normoxia: ~ 21% oxygen; DFX, deferoxamine. Data were presented as the mean ± standard deviation. *P < 0.05, **P < 0.01, and ***P < 0.001

To uncover the mechanisms underlying NXPH4-induced cancer progression, we analyzed associations between NXPH4 expression and pathway scores using the TCGA database. Our findings revealed considerable associations between NXPH4 expression and multiple signaling pathways, encompassing angiogenesis, tumor proliferation, DNA replication, the epithelial–mesenchymal transition (EMT) process, and hypoxia (Fig. 5A; Fig. S13). Notably, the HIF signaling pathway influences various other signaling pathways. Employing HIF reporter plasmids containing hypoxia response elements, our investigations demonstrated increased luciferase activity upon NXPH4 overexpression and a corresponding decrease upon NXPH4 silencing (Fig. 5B–D; Fig. S14). Interestingly, NXPH4 levels remained unchanged under hypoxia, while the protein level of HIF1A displayed an increase with NXPH4 upregulation and a decrease with NXPH4 downregulation (Fig. 5E–G; Fig. S15). Furthermore, NXPH4 exhibited moderate upregulation in HIF1A expression under hypoxic conditions (Fig. 5H–I).

Next, we aimed to identify downstream genes regulated by HIF1A in CRC. We used a comprehensive approach, considering genes correlated with NXPH4 expression in CRC and HCC, genes associated with OS in CRC, and genes demonstrating a two-fold differential expression between tumor and non-tumor tissues in CRC. We identified a core set of 25 genes using Venn diagrams and selected five genes with particularly pronounced differences for further validation (Fig. 5J). Subsequent HIF1A-specific ChIP assays unveiled that HIF1A could regulate ZIC5, FOXD1, TIMP1, HES4, and CCNF, which was further supported by increased luciferase activity under hypoxic or simulated hypoxic conditions, indicating direct regulation of these genes by HIF1A (Fig. 5K, L). Moreover, silencing NXPH4 reduced the enrichment of HIF1A antibody in the promoter regions of ZIC5, FOXD1, TIMP1, HES4, and CCNF (Fig. S16). Transcriptional activity in the promoter regions of these genes was found to be downregulated with NXPH4 silencing (Fig. 5M). In addition, after pretreating cells with hypoxia for 1 week, knocking down NXPH4 significantly reduced cell proliferation under hypoxic conditions (Fig. S17A, B). The mouse subcutaneous tumor model also showed that HIF1A exhibited reduced expression level after NXPH4 knockdown (Fig. S17C).

### NXPH4 stabilized HIF1A via competitively binding to PHD4

**Fig. 6:**
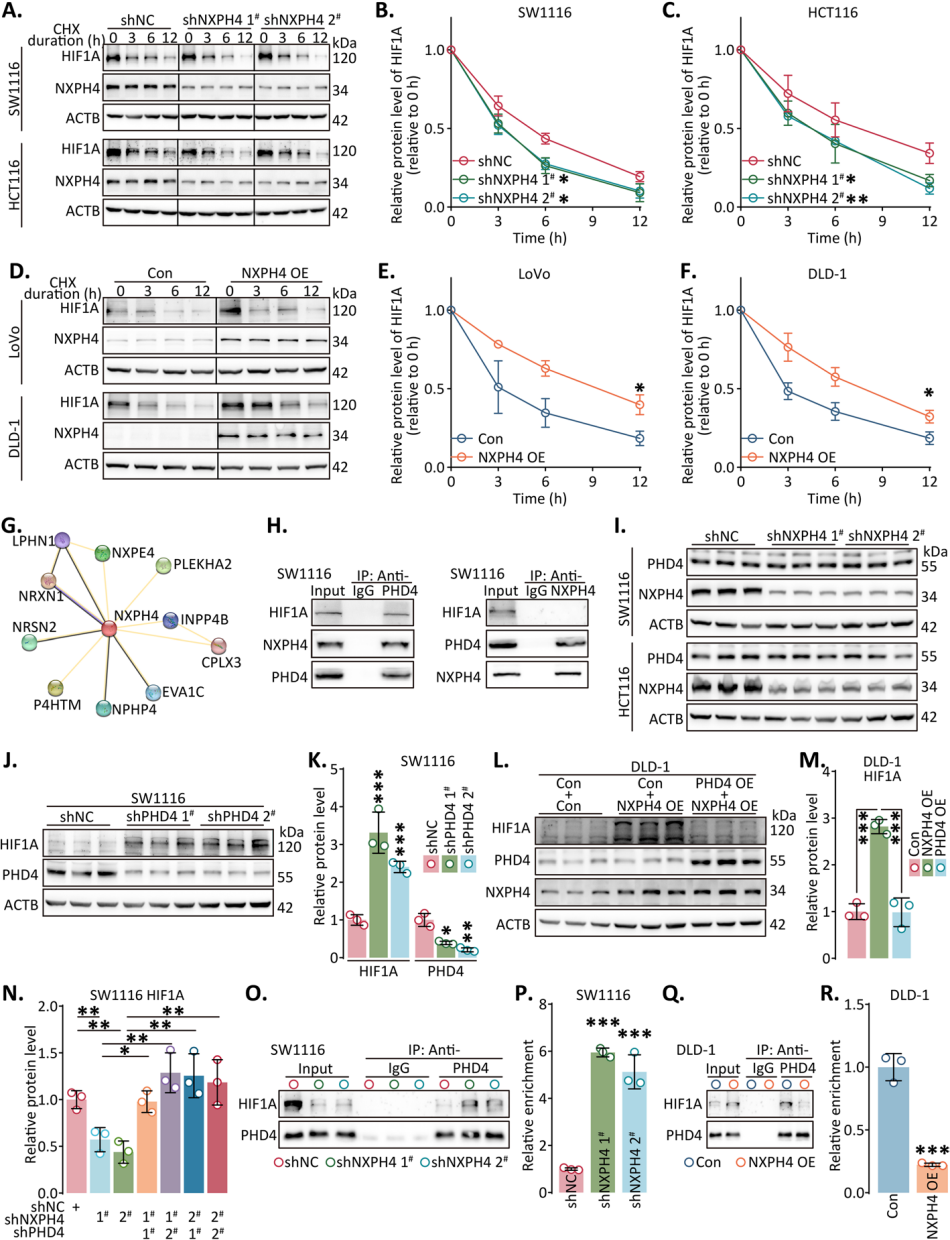
NXPH4 stabilized HIF1A via competitively binding to PHD4. **A–F** As a result of blocking new protein translation with cycloheximide, we performed a western blot to determine the stability of HIF1A following transfection with lentivirus. **G** Potential NXPH4-binding partners were predicted using a STRING database. **H** After lysing the cells, an anti-PHD4 antibody, anti-NXPH4 antibody, or normal rabbit IgG were used for co-IP assay. **I–N** After lentivirus transfection, HIF1A protein level was analyzed using western blot. **O**–**R** The cells transfected with indicated lentivirus were lysed, then anti-PHD4 antibody or normal rabbit IgG were used in co-IP assays. Data were presented as the mean ± standard deviation. *P < 0.05, **P < 0.01, and ***P < 0.001

To explore the precise mechanism underlying the NXPH4-mediated upregulation of HIF1A, we investigated the degradation rate of HIF1A. Our findings revealed an accelerated degradation rate of HIF1A upon NXPH4 knockdown, while its half-life was extended with NXPH4 overexpression (Fig. 6A–F; Fig. S18). Subsequently, we constructed a protein–protein interaction network using established and predicted interactions from the STRING DB database, which suggested a potential interaction between NXPH4 and PHD4, coded by P4HTM (Fig. 6G). As a member of the prolyl hydroxylase domain-containing protein family, PHD4 modulates HIF hydroxylation, thereby influencing cellular adaptation to hypoxia. To validate these interactions, we performed co-immunoprecipitation (co-IP) assays for HIF1A, NXPH4, and PHD4. Interestingly, no direct binding between NXPH4 and HIF1A was observed (Fig. 6H; Fig. S19A).

While NXPH4 did not directly affect PHD4 expression levels, ectopic expression of PHD4 reduced HIF1A protein levels (Fig. 6I; Fig. S19B–F). Conversely, PHD4 knockdown led to an increase in HIF1A protein levels. Furthermore, overexpressing PHD4 attenuated the increased HIF1A levels induced by NXPH4 overexpression, and knocking down PHD4 reversed the HIF1A reduction caused by NXPH4 knockdown (Fig. 6J–N; Fig. S19G–O). Co-IP assays demonstrated a notable increase in the interaction between HIF1A and PHD4 antibodies upon NXPH4 knockdown (Fig. S20). Conversely, NXPH4 upregulation weakened the interaction between PHD4 and HIF1A (Fig. 6O–R). Overall, our findings suggest that NXPH4 stabilizes HIF1A by competitively binding to HIF1A, thus activating the HIF1A signaling pathway.

## Discussion

CRC and HCC are frequently diagnosed malignancies in DSCs with high incidence and mortality rates [4, 28]. However, existing early detection methods are limited and often lead to delayed tumor diagnoses. Moreover, CRC and HCC commonly exhibit recurrences, metastases, and drug resistance, significantly impacting patient prognosis. Consequently, there is an urgent need to explore more effective diagnostic and therapeutic markers to address these challenges. This research demonstrates elevated levels of NXPH4 in CRC and HCC contribute to cancer cell proliferation, growth, and metastasis, underscoring its role in tumor progression.

NXPH4, a member of the NXPH protein family, is closely associated with neuronal cell signal transduction and known for its interaction with Neurexin [12], which regulates synaptic transmission. Emerging research highlights the pivotal roles of NXPH family members, particularly NXPH1 and NXPH4, in influencing the prognosis and advancement of diverse tumors. In neuroblastoma, NXPH1 has been shown to impact tumor growth, metastasis, and patient prognosis [29]. Similarly, NXPH4 regulates cell cycle dynamics and tumor progression in non-small cell lung cancer [15] and bladder cancer [17]. Prior studies have suggested a potential collaborative diagnostic utility of NXPH4 alongside other proteins for identifying CRC [30]. Our findings provide insights into the tumor-promoting effects of NXPH4 in CRC and HCC, highlighting how RNA modifications might influence *NXPH4* mRNA to evade RNautophagy and consequently elevate NXPH4 expression.

RNautophagy is a self-degradative pathway responsible for transporting RNA molecules directly into lysosomes for degradation [11], which involves the participation of both the lysosomal membrane protein LAMP2C and SQSTM1 [27]. LAMP2C’s cytoplasmic domain interacts directly with nucleic acids, facilitating their recruitment and subsequent lysosomal degradation in an ATP-dependent manner [31]. Dysregulated autophagy has been strongly associated with tumorigenesis, progression, and metastasis. Recent evidence has also implicated RNautophagy in the development of chemoresistance to decitabine in gastric cancer, where SQSTM1-mediated degradation of Linc00942 leads to elevated DNA methylation levels via increased DNMT3A expression [32]. In our study, we uncovered the role of SQSTM1 in mediating RNautophagy-driven degradation of NXPH4, particularly in the absence of m^5^C modification.

m^5^C methylation is a prevalent modification found in various types of RNAs [33]. Earlier research has primarily focused on the functional significance of m^5^C methylation in transfer RNA (tRNA) [34], particularly its role in regulating translation accuracy. Recent studies have suggested that similar to m^6^A, m^5^C methylation can also impact mRNA maturation, metabolism, and translation processes [35, 36]. Extensive research has demonstrated the crucial involvement of regulatory factors in m^5^C methylation from biological to pathological processes, including proliferation, differentiation, embryonic development, tumor occurrence, and immunity [33]. The proteins involved in regulating m^5^C methylation can be categorized into three groups: writers, erasers, and readers. For instance, the methylase NSUN2 exhibits significantly higher expression in gastric cancer tissues than non-cancerous tissues, potentially suppressing the expression of p57Kip2 and promoting gastric cancer cell proliferation [37]. Moreover, NSUN2 functions as a glucose sensor, ensuring mRNA stability of three prime repair exonuclease 2 during glucose deficiency, thereby inhibiting the GAS/STING pathway, sustaining tumor development, and conferring resistance to immune therapy [38]. TET2, an enzyme facilitating mRNA demethylation, is implicated in regulating the migration and self-renewal of leukemia stem cells [39]. The reader protein YBX1 recognizes m^5^C-modified mRNA and recruits ELAVL1 to stabilize hepatoma-derived growth factor mRNA, thus facilitating bladder cancer cell growth and metastasis [40]. An upregulation of another reader, ALYREF, under the HIF1A control, contributes to enhanced glucose metabolism in bladder cancer [41].

As transcription factors, HIFs display critical roles in low-oxygen conditions in cells [42]. In cancer, HIF has emerged as a key mechanism contributing to various aspects of tumor biology, including angiogenesis, metabolic reprogramming [43, 44], and tumor progression. HIF activation drives metabolic changes that allow cancer cells to thrive in oxygen-deprived environments, altering energy metabolism, evading apoptosis, and promoting cell survival [45]. Furthermore, HIF activation promotes angiogenesis, the formation of new blood vessels, which provides oxygen and nutrients to support tumor growth and facilitate metastasis [46]. Additionally, HIF activation can confer resistance to chemotherapy and targeted therapies through various pathways [47], including enhanced DNA repair and reduced drug uptake and transport. Moreover, hypoxia suppresses immune responses in tumor cells, allowing them to evade immune system attacks, weakening antitumor immune effects by increasing immune-inhibitory cells and reducing tumor antigen expression [48].

## Conclusion

In summary, we identified NXPH4 as a novel prognostic biomarker and a gene that promotes CRC and HCC tumor progression. Our mechanistic investigations have revealed that m^5^C modification in *NXPH4* mRNA reduces its binding affinity with SQSTM1, thus preventing RNautophagic degradation. Furthermore, the competitive binding of NXPH4 protein with PHD4 impedes HIF1A degradation and activates the HIF signaling pathway. Collectively, this study contributes to a better understanding of the mechanisms associated with CRC pathogenesis and offers potential insights into innovative diagnostic and therapeutic approaches in DSCs.

### Supplementary Information

Supplementary Material 1.

## Data Availability

The datasets used and/or analyzed during the current study are available from the corresponding author on reasonable request.

## Abbreviations

CRC: Colorectal cancer
NXPH4: Neurexophilin 4
HIF1A: Hypoxia inducible factor-1A
PHD4: Prolyl 4-hydroxylase, transmembrane
NSUN4: NOP2/Sun RNA methyltransferase 4
ChIP: Chromatin immunoprecipitation
CoIP: Co-immunoprecipitation
GFP: Green fluorescent protein
gRNA: Guide RNA
m^5^C: 5-Methylcytidine
m^6^A: *N*^6^-Methyladenosine
RIP: RNA immunoprecipitation
