# Supplementary material for: NXPH4 mediated by m5C contributes to the malignant characteristics of colorectal cancer via inhibiting HIF1A degradation
Source: Cell Mol Biol Lett. 2024 Aug 20;29:111. doi: 10.1186/s11658-024-00630-5 (PMC11334498; doi:10.1186/s11658-024-00630-5)

Supplementary Materials for

NXPH4 mediated by m5C contributes to the malignant characteristics of colorectal cancer via inhibiting HIF1A degradation

Lei Yang[1,2#], Jiawen Shi[1#], Mingyang Zhong[1,3#], Pingping Sun[1,2], Xiaojing Zhang[1,2], Zhengyi Lian[1,3], Hang Yin[1], Lijun Xu[1,3], Guyin He[1,3], Haiyan Xu[1], Han Wu[3], Ziheng Wang[4], Kai Miao[4*], Jianfei Huang[1,2*]
# These authors contributed equally to this work.
*Correspondence to: Jianfei Huang: jfhuang@ntu.edu.cn
Kai Miao: kaimiao@um.edu.mo

This PDF file includes:

Figures. S1 to S20
Tables S1 to S6
Original blots

Supplementary Figures

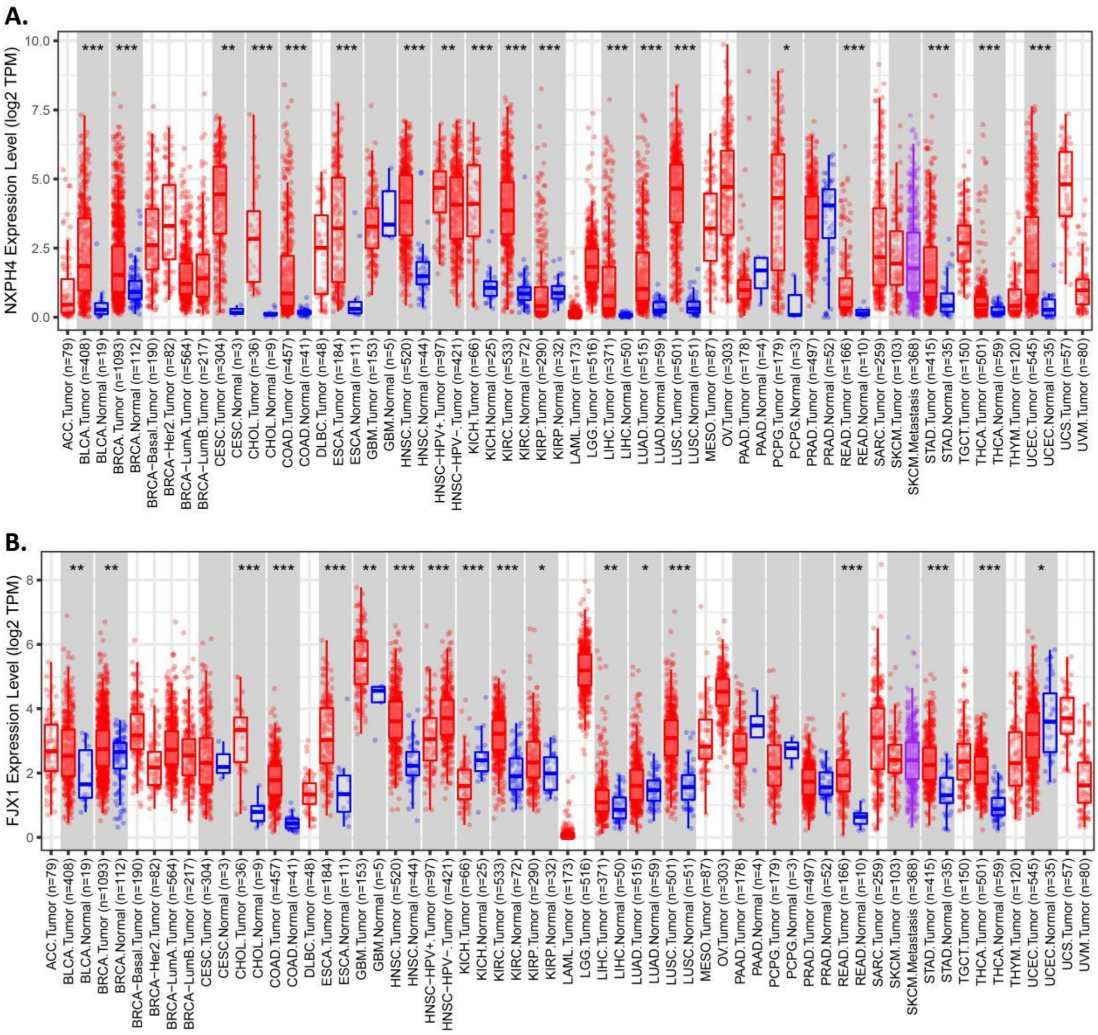

Figure S1 NXPH4 (A) and FJX1 (B) mRNA expression in diverse human malignancies. Statistical significance was calculated using the Wilcoxon test. **P* < 0.05, ** *P* < 0.01, and *** *P* < 0.001

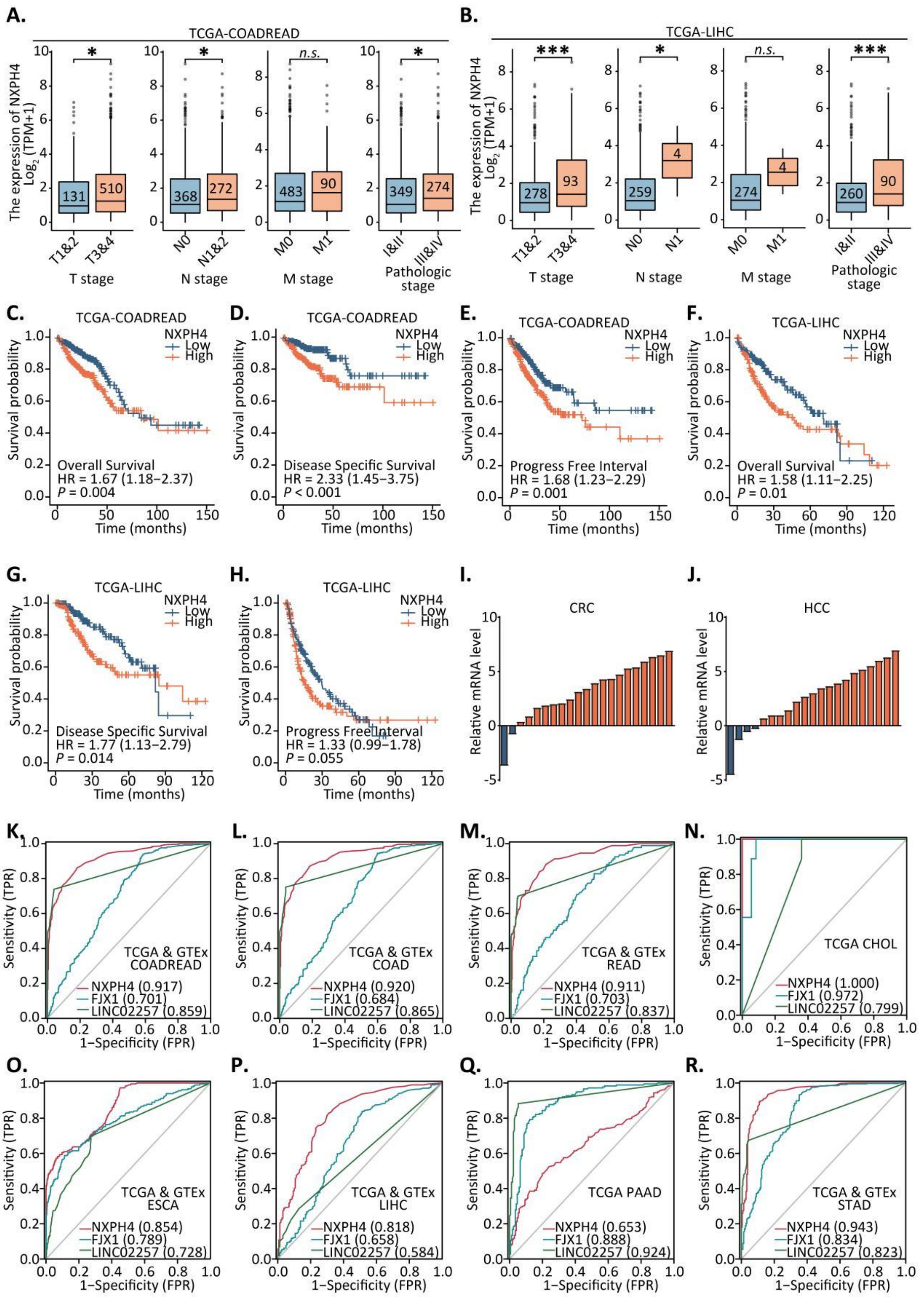

Figure S2 High NXPH4 expression in CRC and HCC was associated with poor prognosis. **(A-B)** NXPH4 mRNA expression was analyzed in different pathological stages for CRC and HCC in the TCGA database. **(C-H)** High NXPH4 expression was correlated with poor overall survival (OS), disease-specific survival (DSS), and progression-free interval (PFI) in CRC and HCC. **(I-J)** qPCR analysis of relative

VSX1 mRNA expression in 21 pairs of human clinical CRC or HCC tissues. **(K-R)** NXPH4, FJX1, and LINC02257 showed a high accuracy in predicting normal and cancer outcomes via ROC curves. *$P < 0.05$, ** $P < 0.01$, and *** $P < 0.001$, n.s. = no significance

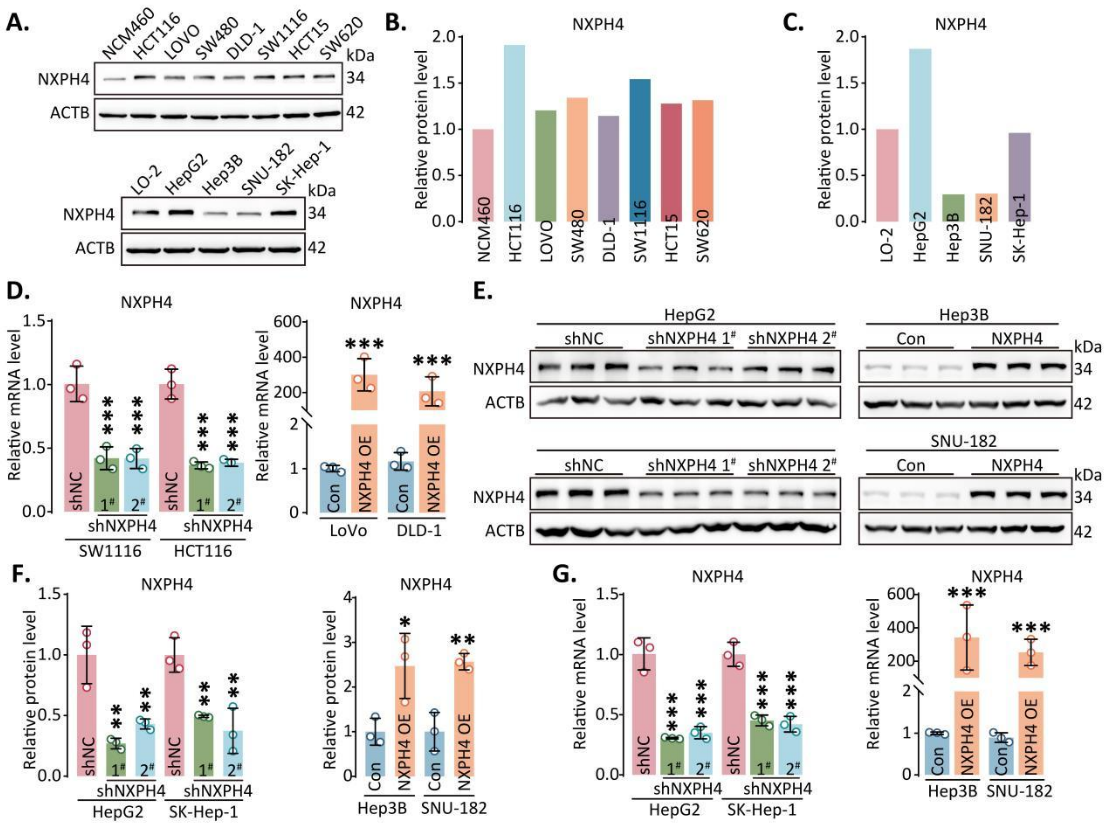

Figure S3 Confirmation of NXPH4 overexpression or knockdown. (A-C) NXPH4 protein expression levels in a panel of human CRC and HCC cell lines were detected by western blot. **(D)** Western blot verified the knockdown of NXPH4 in SW1116 and HCT116 cells and the overexpression of NXPH4 in LoVo and DLD-1 cells. **(E-F)** Western blot verified the knockdown of NXPH4 in HepG2 and SK-Hep-1 cells and the overexpression of NXPH4 in Hep3B and SNU-182 cells. **(G-H)** qPCR verified the overexpression of NXPH4 and the knockdown of NXPH4 in CRC and HCC cells. Data were presented as the mean ± standard deviation. *$P < 0.05$, ** $P < 0.01$, and *** $P < 0.001$

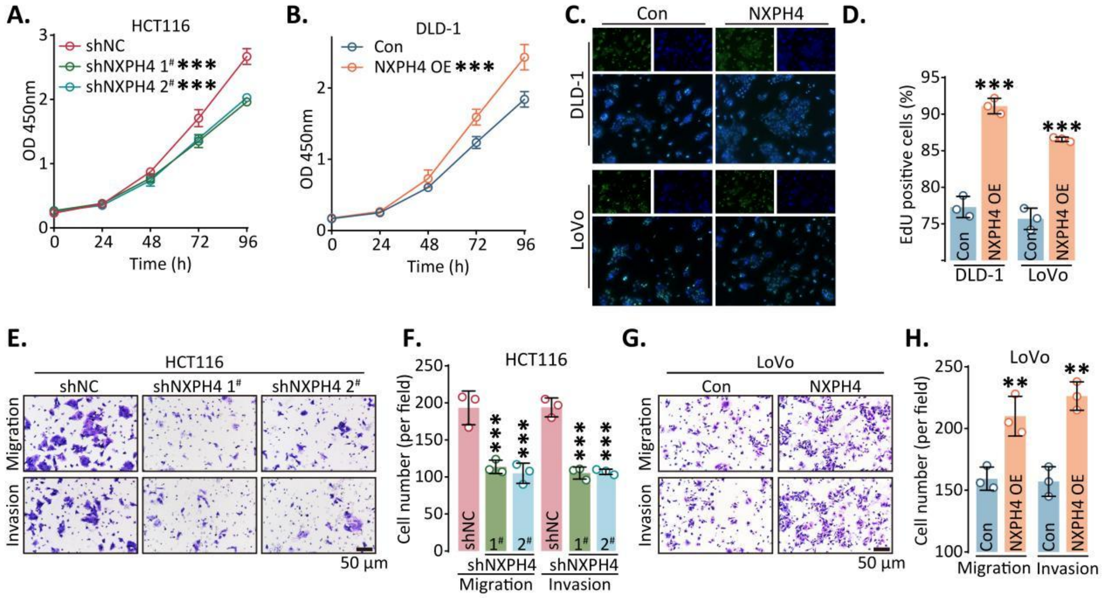

Figure S4 High NXPH4 expression promoted CRC cell proliferation, invasion, and migration. (A-B) CCK-8 evaluated cell proliferation following NXPH4 knockdown or overexpression. **(C-D)** EdU assay determined the capacity of DNA synthesis following NXPH4 upregulation. **(E-H)** Transwell assay evaluated cell invasion and migration following NXPH4 knockdown or overexpression. Data were presented as the mean ± standard deviation. ** $P < 0.01$, and *** $P < 0.001$

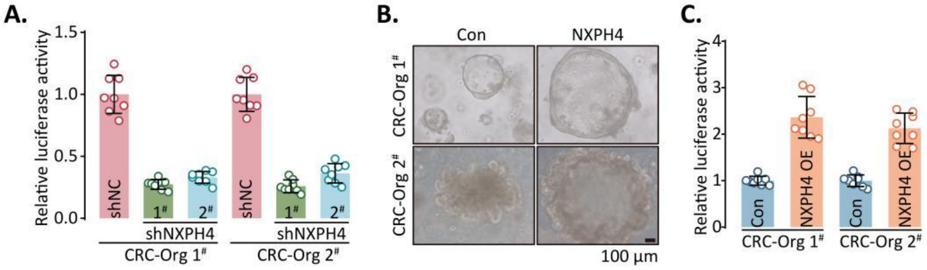

Figure S5 High NXPH4 expression promoted CRC organoids proliferation. (A) Statistical results of the luciferase activity in each group of CRC organoids. **(B)** Representative pictures of CRC organoids are shown following NXPH4 overexpression. **(C)** Statistical results of the luciferase activity in each group of CRC organoids. Data were presented as the mean ± standard deviation.

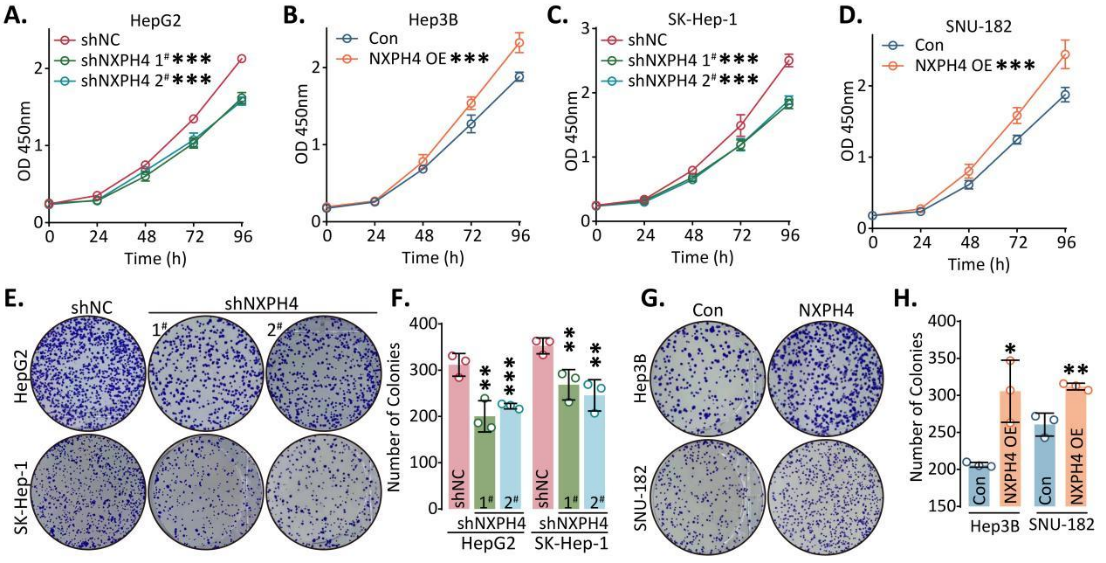

Figure S6 High NXPH4 expression promoted HCC cell proliferation. (A-D) CCK-8 evaluated cell proliferation following NXPH4 knockdown or overexpression. **(E-H)** CCK-8 assay evaluated cell capacity of clone formation following NXPH4 knockdown or overexpression. Data were presented as the mean ± standard deviation. * $P < 0.05$, ** $P < 0.01$, and *** $P < 0.001$

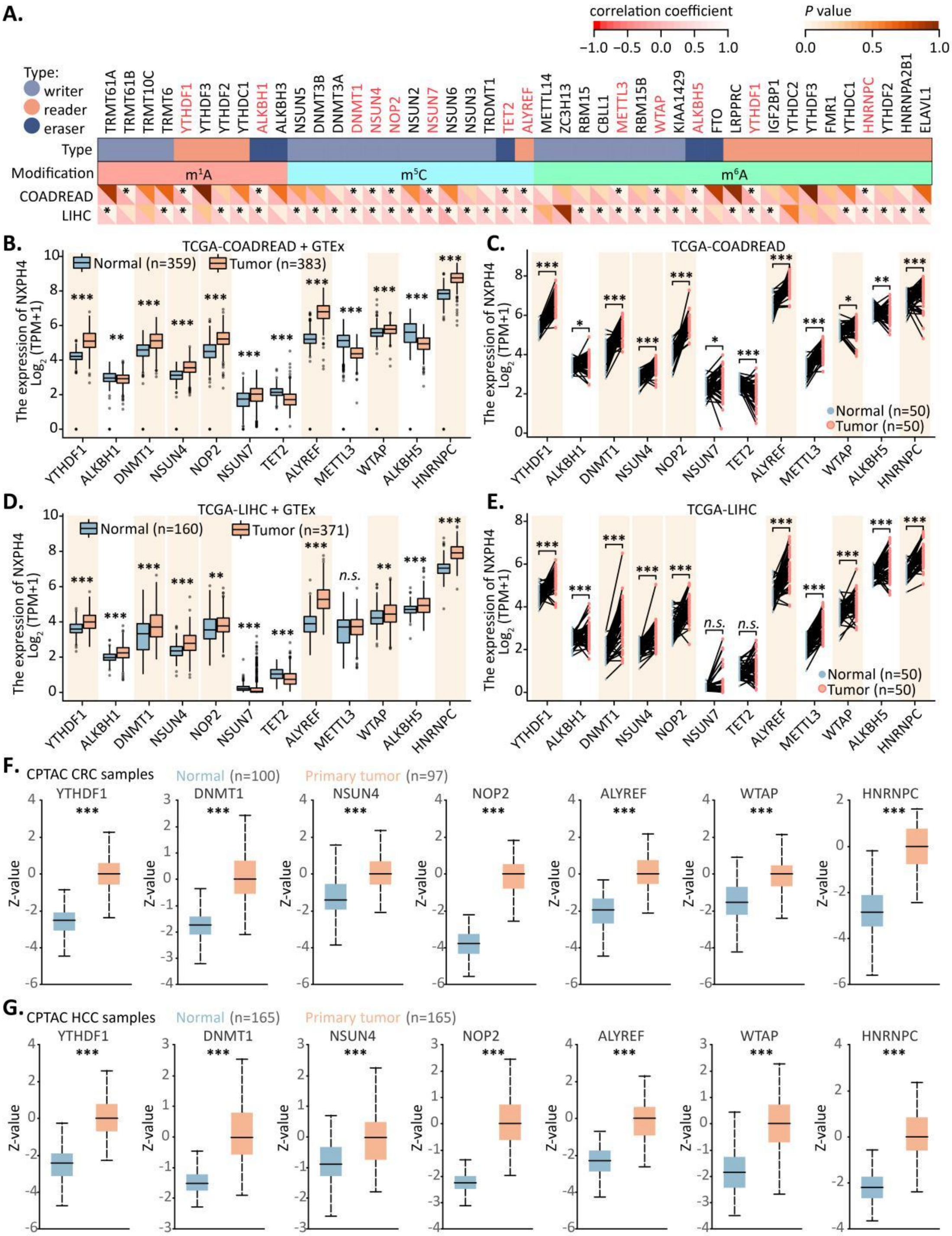

Figure S7 Gene correlation analyses suggested that NXPH4 might be regulated by RNA modification. **(A)** Gene correlation analyses between NXPH4 and genes associated with RNA modification in CRC and HCC from TCGA via Spearman's correlation coefficient. **(B)** mRNA expression of candidate genes in CRC. Statistical significance was calculated using the Wilcoxon test. **(C)** mRNA expression of candidate genes was determined in tumor tissue and adjacent normal tissue. The significance of the difference was evaluated using the paired Student's *t*-test. **(D)** mRNA expression of candidate genes in HCC. Statistical significance was calculated

using the Wilcoxon test. **(E)** mRNA expression of candidate genes was determined in tumor tissue and adjacent normal tissue. The significance of the difference was evaluated using the paired Student's *t*-test. **(F-G)** Protein expression of candidate genes in CRC and HCC from CPTAC. Statistical significance was calculated using the Wilcoxon test. Data were presented as the mean ± standard deviation. *$P < 0.05$, ** $P < 0.01$, and *** $P < 0.001$, n.s. = no significance

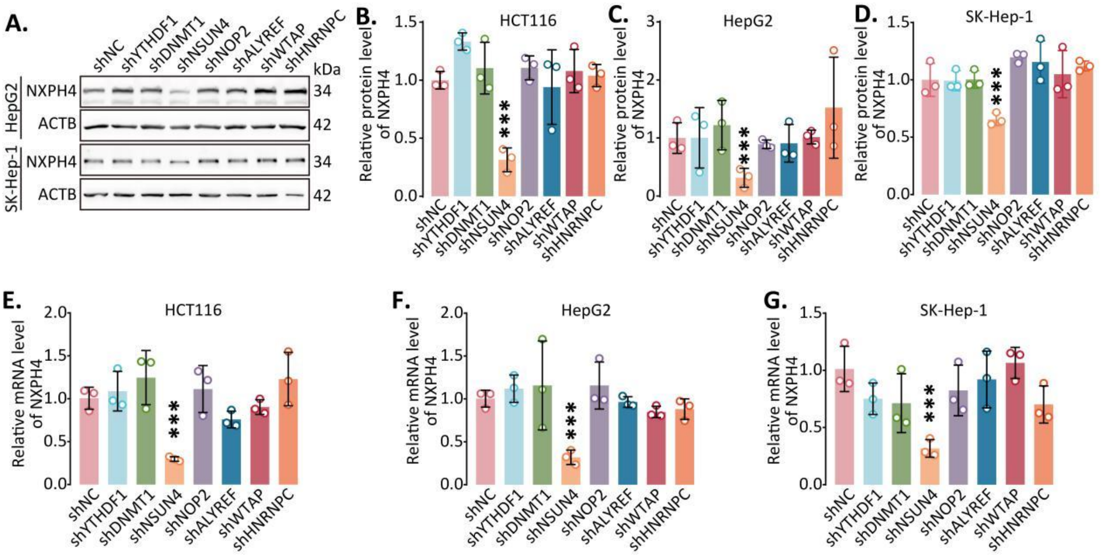

Figure S8 NXPH4 might be upregulated by NSUN4. Western blot and qPCR analysis of expression of NXPH4 in HCT116, HepG2, and SK-Hep-1 cells following transfection with indicated lentivirus. Data were presented as the mean ± standard deviation. *** $P < 0.001$

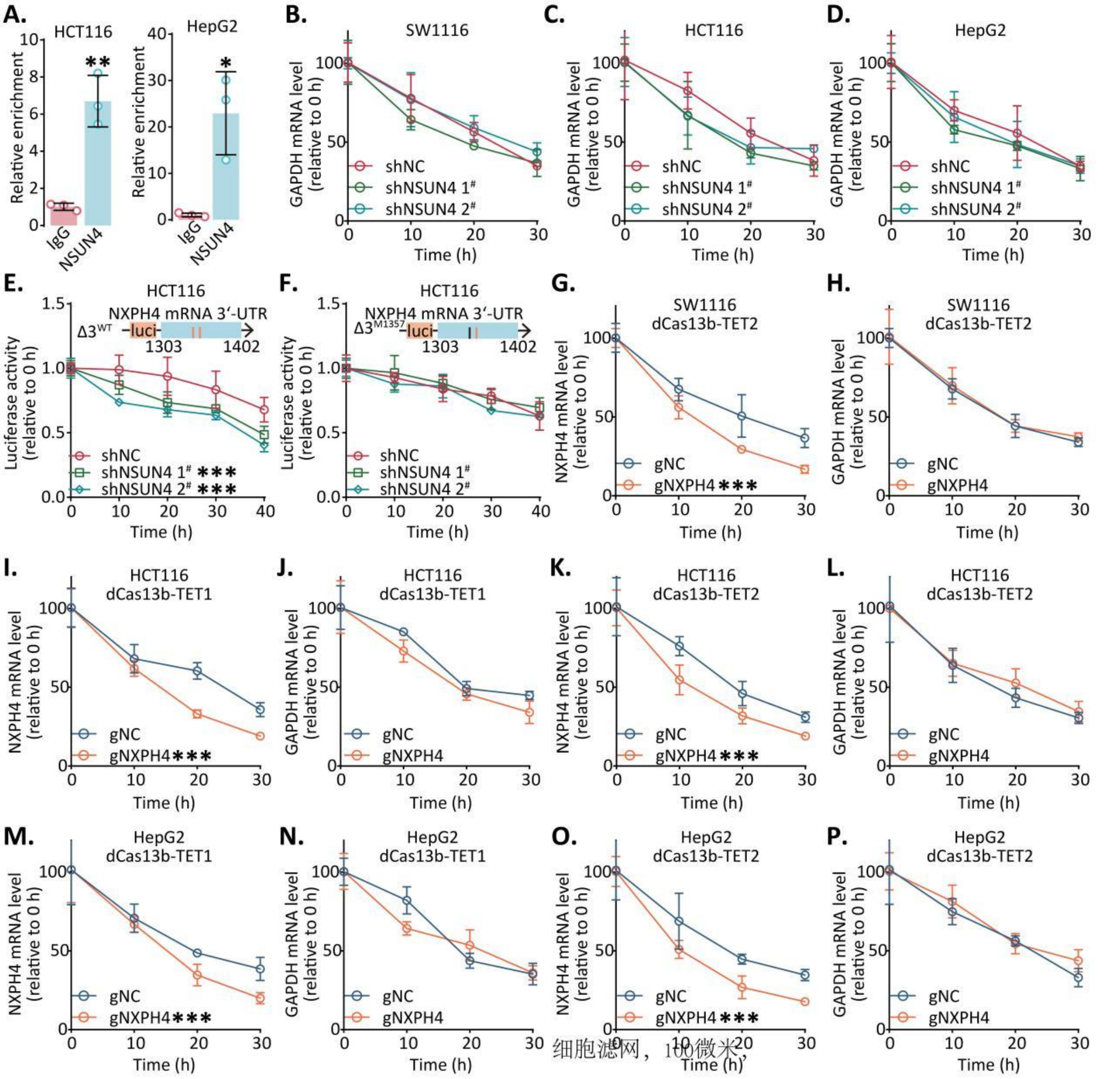

Figure S9 NXPH4 could be increased by NSUN4 via m^{5}C modification. (A) NXPH4 mRNA enrichment was assessed by RIP assay and qPCR, and the mRNA enrichment was quantified by IgG control. **(B-D)** qPCR was performed to determine the stability of GAPDH mRNA in after transfection with NSUN4-shRNA relative to 0 hour after blocking new RNA transcription with α-amanitin. **(E-F)** Luciferase activity of NXPH4-full-length and the NXPH4-mutation was measured after being treated with indicated lentivirus. **(G-P)** qPCR was performed to assessed the stability of NXPH4 mRNA and GAPDH mRNA following transfection indicated lentivirus. Data were presented as the mean ± standard deviation. *$P < 0.05$, ** $P < 0.01$, and *** $P < 0.001$, n.s. = no significance

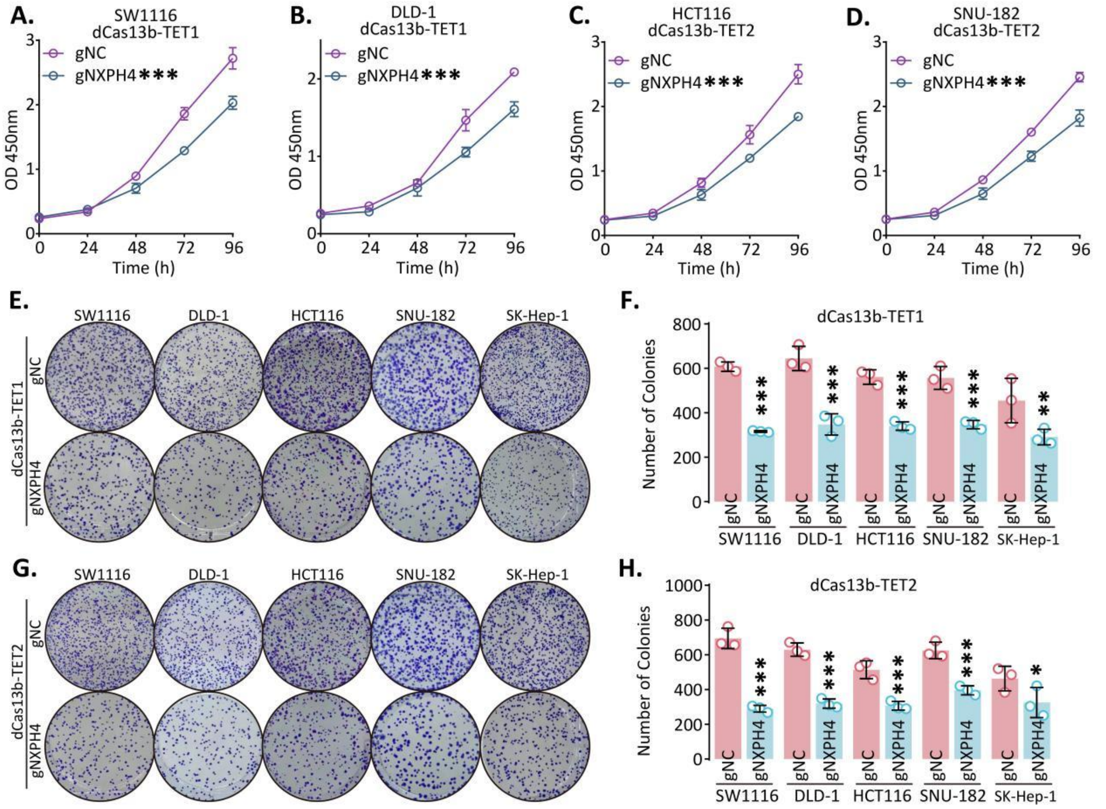

Figure S10 m^{5}C increase the level of NXPH4 to accelerated proliferation of CRC and HCC. (A-D) CCK-8 evaluated cell proliferation following transfection with indicated lentivirus. **(E-H)** Removing the m^{5}C modification in NXPH4 mRNA inhibited colony formation. Data were presented as the mean ± standard deviation. **P* < 0.05, ** *P* < 0.01, and *** *P* < 0.001

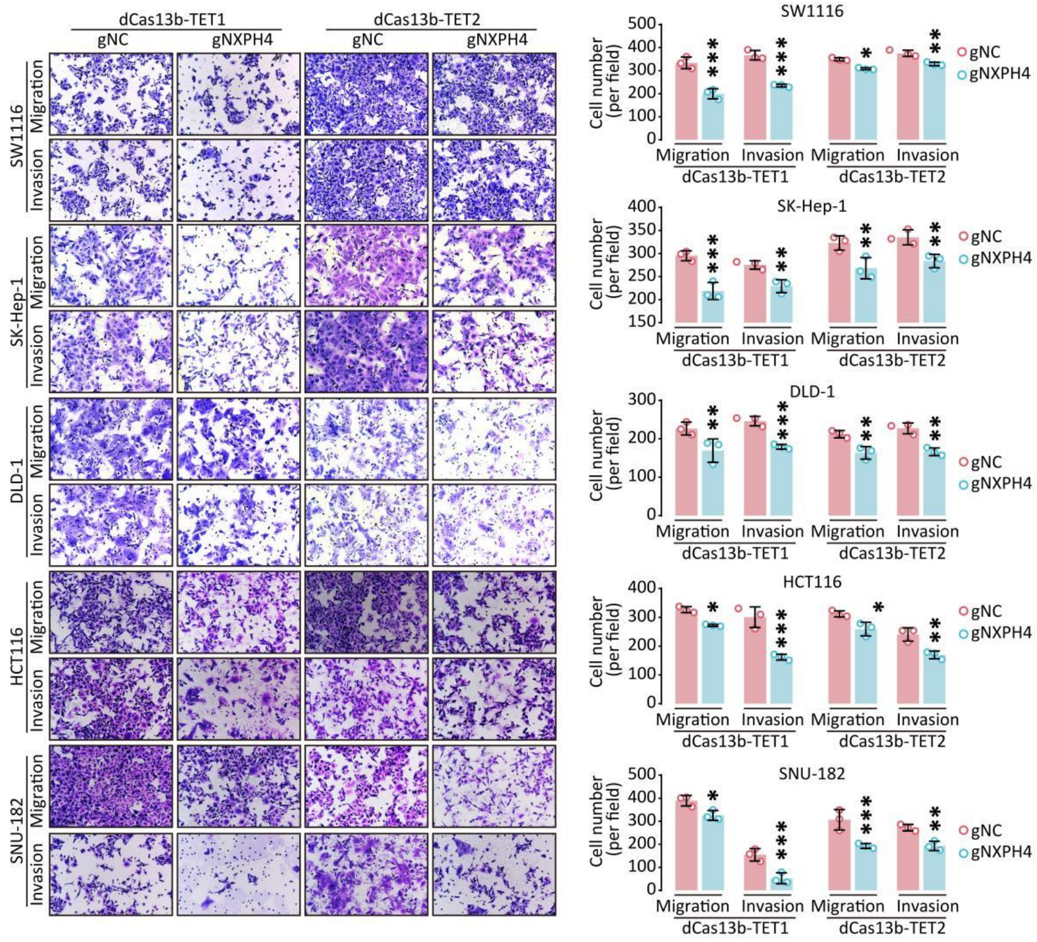

Figure S11 m^{5}C increase the level of NXPH4 to accelerated migration and invasion of CRC and HCC. Transwell assay evaluated cell invasion and migration following transfection with indicated lentivirus. Data were presented as the mean ± standard deviation. *$P < 0.05$, ** $P < 0.01$, and *** $P < 0.001$

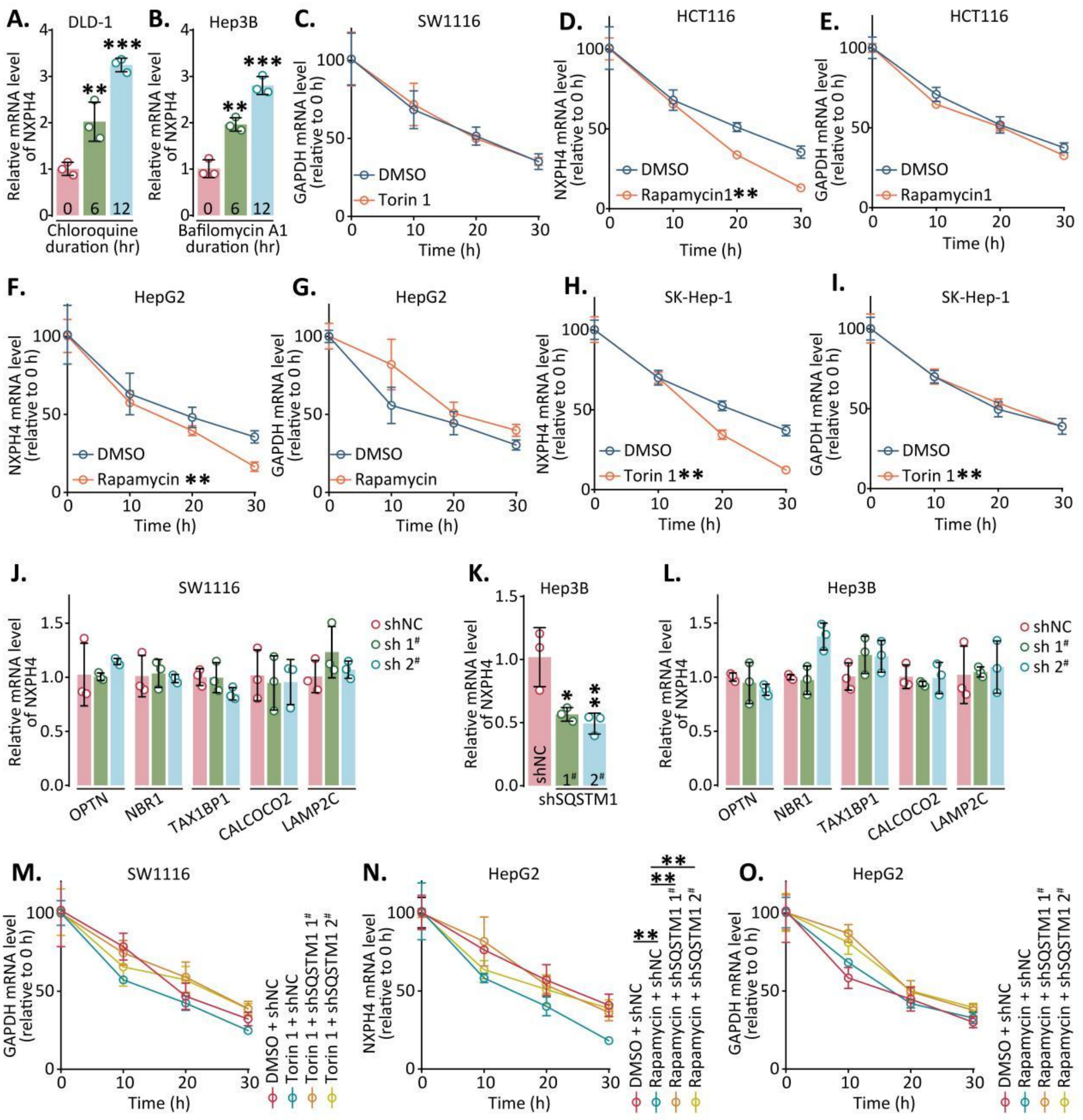

Figure S12 RNautophagy might regulated the expression of NXPH4 in m^{5}C. (A) NXPH4 mRNA expression levels were detected by qPCR after treatment of Chloroquine or Bafilomycin A1. **(C-I)** qPCR was performed to assessed the stability of NXPH4 mRNA and GAPDH mRNA following treatment of Torin 1 or Rapamycin. **(J-L)** NXPH4 mRNA levels were detected by qPCR after transfection with indicated shRNAs. **(M-O)** qPCR was performed to assessed the stability of NXPH4 mRNA and GAPDH mRNA following indicated treatment. *$P < 0.05$, ** $P < 0.01$, and *** $P < 0.001$

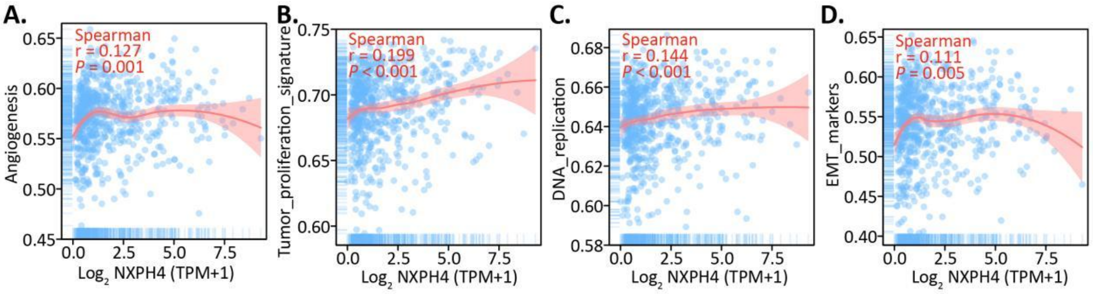

Figure S13 Signaling pathway enrichment analysis of NXPH4.

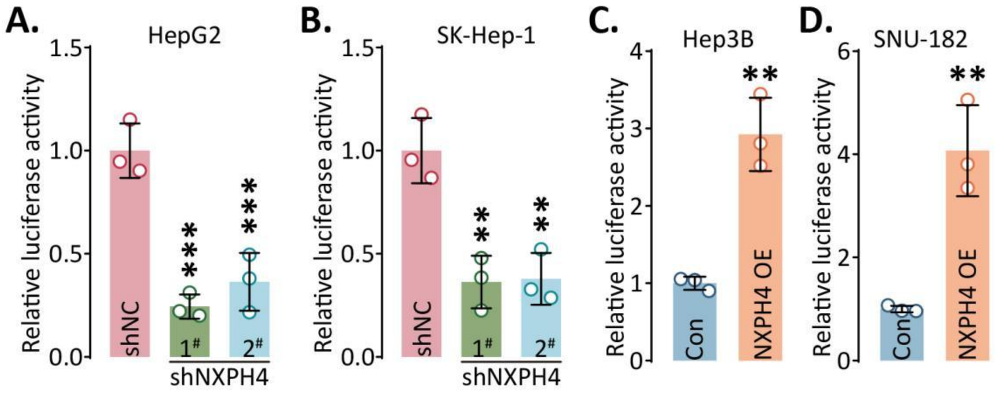

Figure S14 NXPH4 could enhance the activity of HIF signaling pathway. (A-D) Luciferase activity of HIF reporter was measured after being treated with indicated lentivirus. Data were presented as the mean ± standard deviation. ** $P < 0.01$, and *** $P < 0.001$

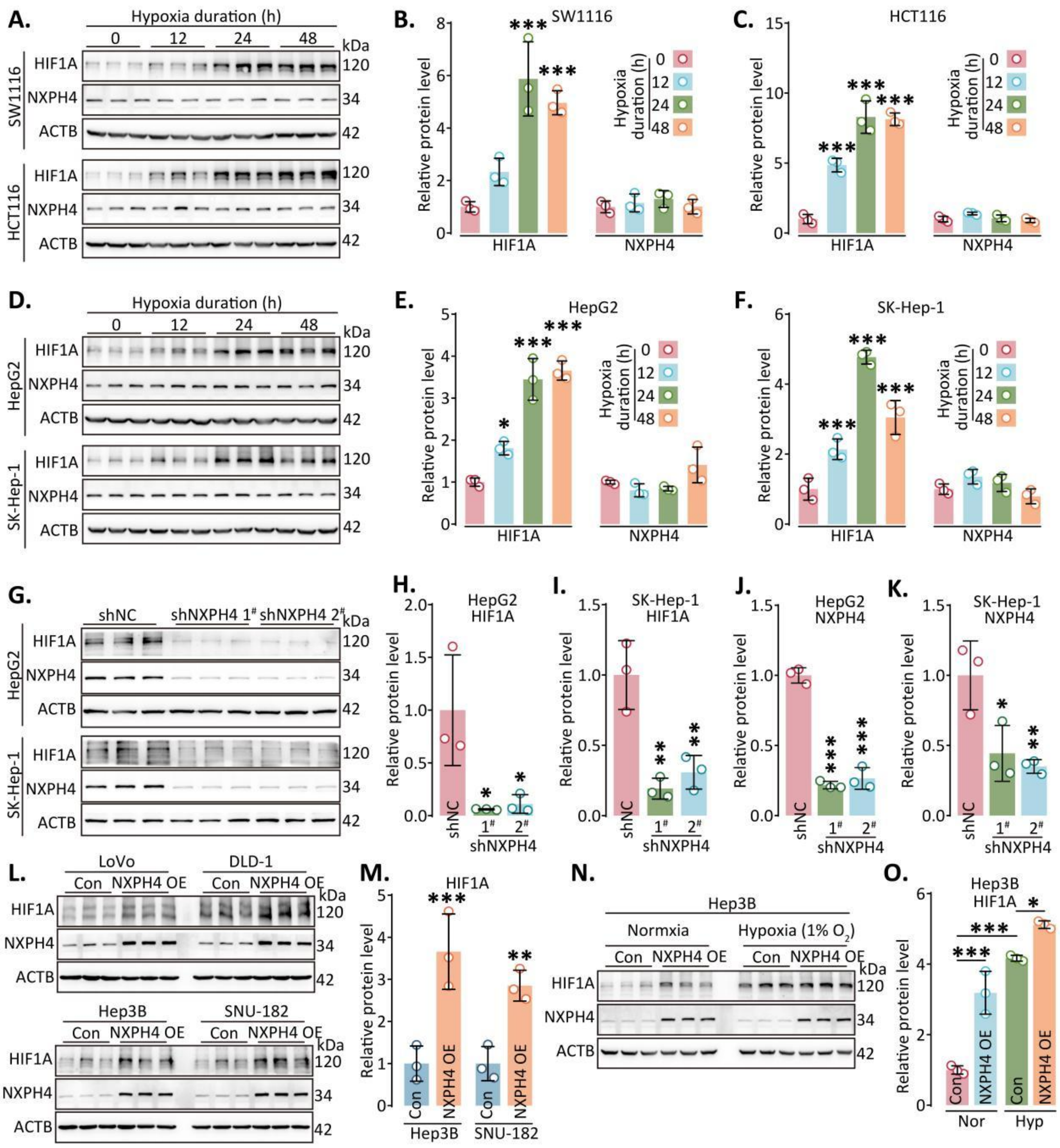

Figure S15 NXPH4 upregulated the protein level of HIF1A. (A-O) Western blot analysis of expression of NXPH4 and HIF1A in CRC and HCC cells following transfection with indicated lentivirus or treatment. Data were presented as the mean ± standard deviation. *$P < 0.05$, ** $P < 0.01$, and *** $P < 0.001$

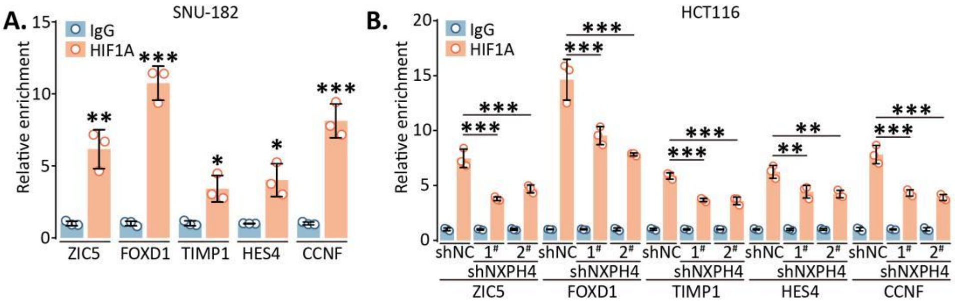

Figure S16 NXPH4 silence inhibited the binding between HIF1A and the downstream genes. (A) ChIP assays showed HIF1A binding to the promoter region of potential target genes. **(B)** ChIP and qPCR assays showed HIF1A binding to the promoter region of potential target genes. Data were presented as the mean ± standard deviation. ** $P < 0.01$ and *** $P < 0.001$

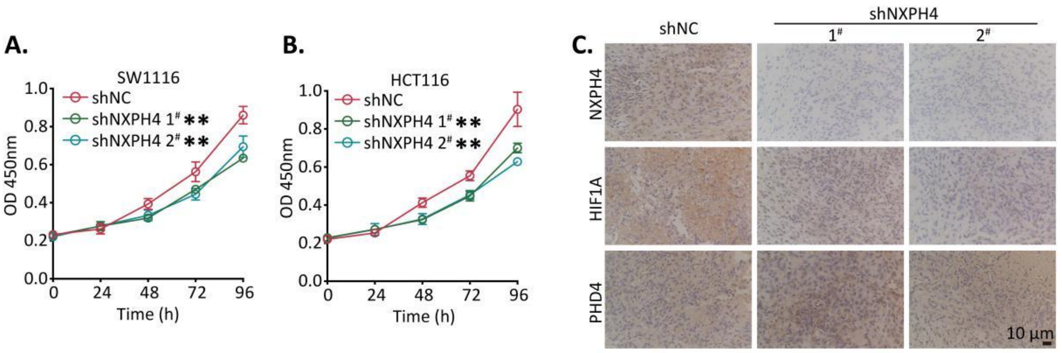

Figure S17 NXPH4 silencing inhibited proliferation in CRC. (A-B) CCK-8 evaluated cell proliferation following transfection with indicated lentivirus under hypoxia. **(C)** Immunohistochemical experiments were used to detect the protein levels of HIF1A, NXPH4, and PHD4 in mouse tumor tissues. Data were presented as the mean ± standard deviation. ** $P < 0.01$

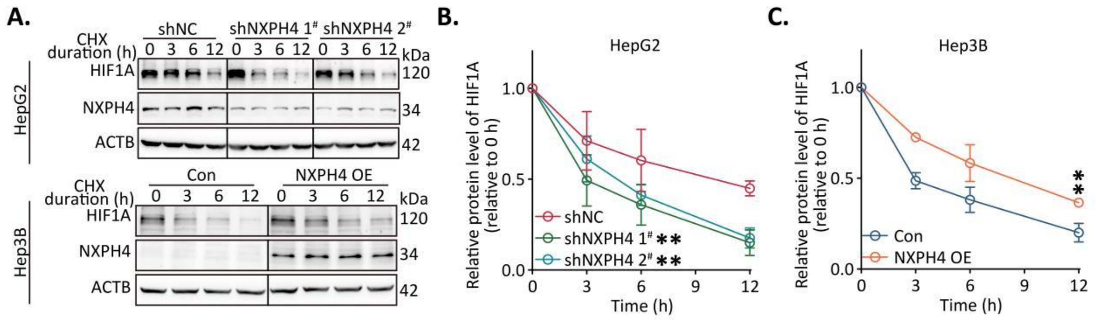

Figure S18 NXPH4 could stabilize HIF1A. (A-C) Western blot was performed to assessed the stability of HIF1A following transfection indicated lentivirus relative to 0 hour after blocking new protein translation with Cycloheximide. Data were presented as the mean ± standard deviation. ** $P < 0.01$

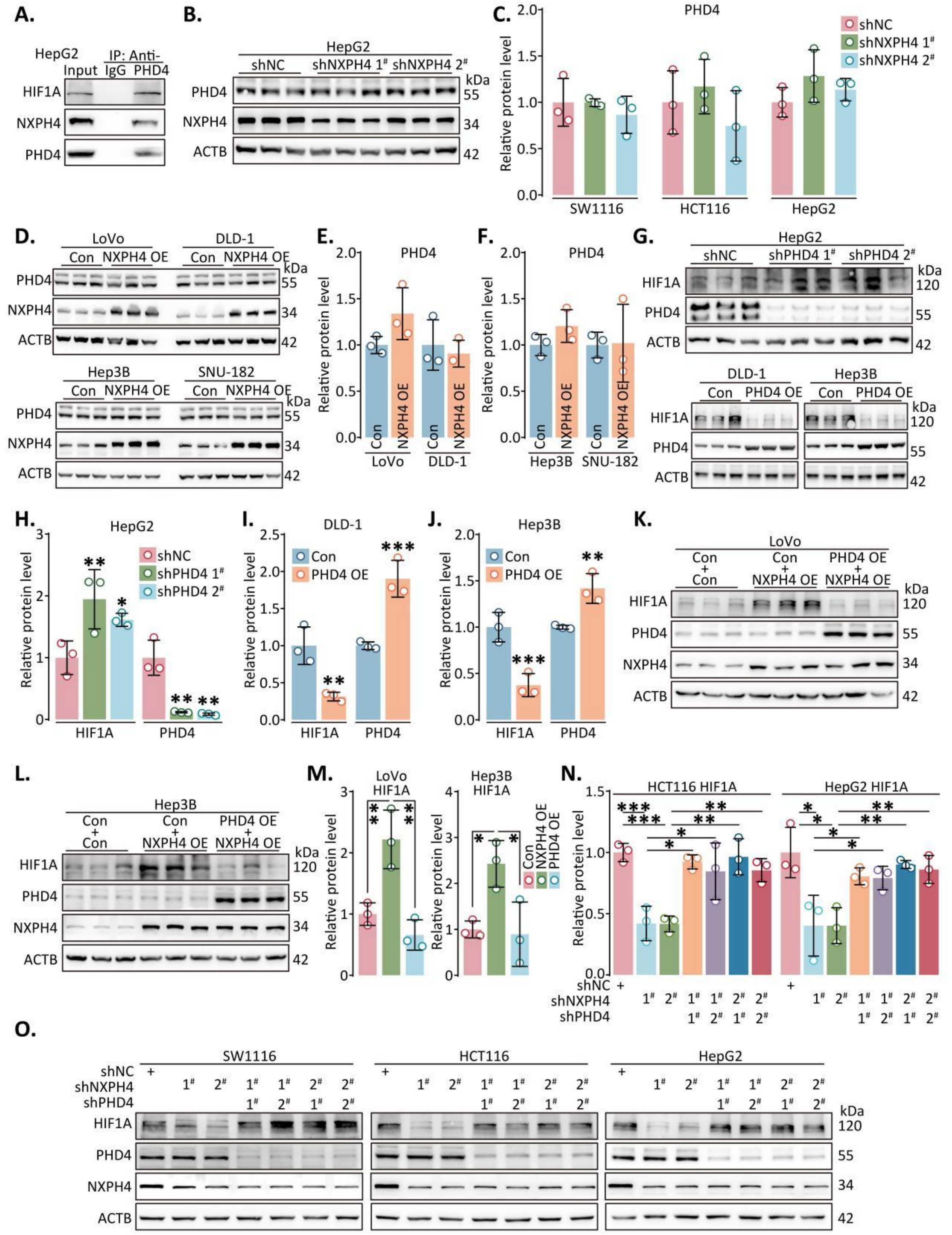

Figure S19 NXPH4 could stabilize HIF1A via PHD4. (A) Cells were lysed, then CoIP assay was performed with anti-PHD4 antibody or normal rabbit IgG. **(B-O)** Western blot was performed to assessed the stability of HIF1A following transfection indicated lentivirus relative to 0 hour after blocking new protein translation with Cycloheximide. Data were presented as the mean ± standard deviation. *$P < 0.05$, ** $P < 0.01$, and *** $P < 0.001$

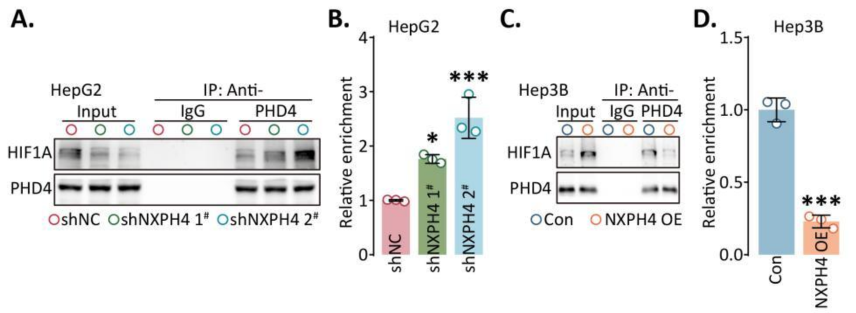

Figure S20 NXPH4 stabilized HIF1A via binding to PHD4. (A-D) Cells transfected with indicated lentivirus were lysed, then CoIP assay was performed with anti-PHD4 antibody or normal rabbit IgG. Data were presented as the mean ± standard deviation. **P* < 0.05 and *** *P* < 0.001

Supplementary Tables

Table S1. The statistical results of basic information.

No.	NXPH4 expression (1: High, 0:Low)	Post-operative survival time (months)	Post-operative survival time (years)	5-year overall survival rate(1:death, 0:Live)	T Stage	T Stage (0: Tis-T1-T2, 1: T3-T4)	N Stage	N Stage (0: N0 , 1: N1-N2)	M Stage	TNM Stage	TMN Stage (0: 0-I-II, 1: III-IV)	Gender(1:Male, 0: Female)	Age	Age (1:≥60, 0:＜60)	Tumor Site (0: Colon, 1: Rectum)	Differentiation (0: Well and Middle, 1: poor)
1	0	70.6	5.88	0	1	0	0	0	0	1	0	1	75	1	0	
2	0	67.2	5.60	0	1	0	0	0	0	1	0	1	42	0	0	0
3	0	63.4	5.28	0	1	0	0	0	0	1	0	0	71	1	1	0
4	0	19.23	1.60	1	1	0	0	0	0	1	0	1	58	0	1	0
5	0	65.4	5.45	0	1	0	0	0	0	1	0	1	72	1	1	0
6	0	67.7	5.64	0	2	0	0	0	0	1	0	0	48	0	0	0
7	0	57.4	4.78	1	2	0	0	0	0	1	0	0	56	0	0	0
8	0	66.4	5.53	1	2	0	0	0	0	1	0	1	77	1	1	0
9	0	60.1	5.01	0	2	0	0	0	0	1	0	1	69	1	0	0
10	0	64.9	5.41	0	2	0	0	0	0	1	0	1	53	0	0	0
11	0	64.2	5.35	0	2	0	0	0	0	1	0	1	58	0	0	0
12	0	100.5	8.38	0	2	0	0	0	0	1	0	0	64	1	0	0
13	0	71.66	5.97	0	2	0	0	0	0	1	0	1	71	1	1	0
14	0	63.3	5.28	0	2	0	0	0	0	1	0	0	73	1	1	0
15	0	65	5.42	0	2	0	0	0	0	1	0	1	76	1	0	0
16	0	62.4	5.20	0	2	0	0	0	0	1	0	0	73	1	1	0
17	0	66.7	5.56	0	2	0	0	0	0	1	0	1	50	0	1	0
18	0	64.1	5.34	0	2	0	0	0	0	1	0	1	79	1	1	0
19	0	58.7	4.89	0	2	0	0	0	0	1	0	1	84	1	0	0
20	0	74.9	6.24	0	2	0	0	0	0	1	0	1	53	0	0	0
21	0	63.3	5.28	0	2	0	0	0	0	1	0	1	66	1	1	0
22	0	62.5	5.21	0	2	0	0	0	0	1	0	0	70	1	0	0
23	0	63.8	5.32	0	2	0	0	0	0	1	0	0	50	0	0	0
24	0	62.2	5.18	0	2	0	0	0	0	1	0	0	85	1	0	0
25	0	94.7	7.89	0	2	0	0	0	0	2	0	1	38	0	0	0
26	0	42.76	3.56	1	3	1	0	0	1	4	1	1	57	0	0	0
27	0	70.7	5.89	0	3	1	0	0	0	2	0	0	65	1	0	0
28	0	63.6	5.30	0	3	1	0	0	0	2	0	1	47	0	0	0
29	0	74	6.17	0	3	1	0	0	0	2	0	0	59	0	0	0
30	0	62.7	5.23	0	3	1	0	0	0	2	0	0	76	1	0	0
31	0	66	5.50	0	3	1	0	0	0	2	0	1	70	1	1	0
32	0	50	4.17	1	3	1	0	0	0	2	0	1	72	1	0	0
33	0	62	5.17	0	3	1	0	0	0	2	0	0	78	1	0	0
34	0	56.1	4.68	1	3	1	0	0	0	2	0	1	90	1	1	0
35	0	39.9	3.33	1	3	1	0	0	0	2	0	0	48	0	1	0
36	0	4	0.33	1	3	1	0	0	0	2	0	1	67	1	0	0
37	0	63.6	5.30	0	3	1	0	0	0	2	0	1	47	0	0	0
38	0	72.9	6.08	0	3	1	0	0	0	2	0	0	47	0	0	0
39	0	30	2.50	1	3	1	0	0	0	2	0	1	53	0	0	
40	0	67	5.58	0	3	1	0	0	0	2	0	1	71	1	0	0
41	0	29.73	2.48	1	3	1	0	0	0	2	0	1	74	1	0	0
42	0	96	8.00	0	3	1	0	0	0	2	0	0	61	1	0	0
43	0	49.93	4.16	1	3	1	0	0	0	2	0	0	74	1	0	0
44	0	62.5	5.21	0	3	1	0	0	0	2	0	0	87	1	0	0
45	0	59	4.92	0	3	1	0	0	0	2	0	1	73	1	0	0
46	0	58.2	4.85	1	3	1	0	0	0	2	0	0	76	1	0	0
47	0	96.2	8.02	0	3	1	0	0	0	2	0	0	74	1	0	0
48	0	0.9	0.08	1	3	1	0	0	0	2	0	0	72	1	0	0
49	0	67.4	5.62	0	3	1	0	0	0	2	0	1	73	1	0	0
50	0	90.4	7.53	0	3	1	0	0	0	2	0	0	68	1	0	0
51	0	91	7.58	1	2	0	1	1	0	3	1	0	54	0	0	
52	0	59.6	4.97	0	2	0	1	1	0	3	1	1	68	1	1	0
53	0	39.5	3.29	1	3	1	1	1	0	3	1	1	59	0	0	0
54	0	62.8	5.23	0	3	1	1	1	0	3	1	0	57	0	1	0
55	0	63.8	5.32	0	3	1	1	1	0	3	1	1	53	0	1	0
56	0	63.5	5.29	0	3	1	1	1	0	3	1	1	73	1	0	0
57	0	35.23	2.94	1	3	1	1	1	0	3	1	0	73	1	1	0
58	0	74.1	6.18	0	3	1	1	1	0	3	1	1	61	1	0	0
59	0	65.4	5.45	0	3	1	1	1	0	3	1	1	71	1	0	0
60	0	51.13	4.26	1	3	1	1	1	0	3	1	1	75	1	0	0
61	0	60	5.00	0	3	1	1	1	0	3	1	0	63	1	0	0
62	0	28.06	2.34	1	3	1	1	1	0	3	1	1	64	1	0	0
63	0	68	5.67	0	3	1	1	1	0	3	1	0	63	1	1	0
64	0	65.2	5.43	0	3	1	1	1	0	3	1	0	81	1	0	
65	0	20.16	1.68	1	3	1	1	1	0	3	1	1	45	0	1	0
66	0	60.4	5.03	0	3	1	1	1	0	3	1	0	68	1	0	0
67	0	64.7	5.39	0	3	1	1	1	0	3	1	1	65	1	1	0
68	0	69	5.75	0	3	1	1	1	0	3	1	1	73	1	1	0

69	0	63	5.25	0	3	1	1	1	0	3	1	1	41	0	0	0
70	0	66.9	5.58	0	3	1	1	1	0	3	1	1	34	0	1	0
71	0	68.3	5.69	0	3	1	2	1	0	3	1	1	60	1	1	0
72	0	66.36	5.53	0	3	1	2	1	0	3	1	1	72	1	0	
73	0	70	5.83	0	3	1	2	1	0	3	1	1	51	0	0	
74	0	31.7	2.64	1	3	1	2	1	0	3	1	0	51	0	0	0
75	0	35	2.92	1	3	1	2	1	0	3	1	1	47	0	0	1
76	0	74.2	6.18	0	3	1	2	1	0	3	1	1	76	1	0	1
77	0	70.76	5.90	0	3	1	0	0	0	2	0	1	69	1	0	0
78	1	68.3	5.69	0	1	0	0	0	0	1	0	0	65	1	1	0
79	1	63.8	5.32	0	2	0	0	0	0	1	0	0	50	0	0	0
80	1	88.7	7.39	0	2	0	0	0	0	1	0	1	59	0	0	0
81	1	63.6	5.30	0	2	0	0	0	0	1	0	1	70	1	1	0
82	1	43.5	3.63	1	2	0	0	0	0	1	0	1	70	1	0	0
83	1	66.7	5.56	0	2	0	0	0	0	1	0	1	58	0	1	0
84	1	66	5.50	0	2	0	0	0	0	1	0	1	59	0	0	0
85	1	64	5.33	0	2	0	0	0	0	1	0	0	60	1	0	0
86	1	69.2	5.77	0	2	0	0	0	0	1	0	1	61	1	1	0
87	1	81.1	6.76	0	2	0	0	0	0	1	0	0	57	0	0	0
88	1	62.5	5.21	0	2	0	0	0	0	1	0	0	78	1	0	0
89	1	68.7	5.73	1	2	0	0	0	0	1	0	1	59	0	1	0
90	1	49.5	4.13	0	2	0	0	0	0	1	0	1	68	1	0	0
91	1	65.4	5.45	0	2	0	0	0	0	1	0	0	78	1	1	0
92	1	14.56	1.21	1	3	1	0	0	0	2	0	1	79	1	1	0
93	1	74.4	6.20	0	3	1	0	0	0	2	0	1	78	1	0	0
94	1	30	2.50	1	3	1	0	0	0	2	0	0	76	1	0	0
95	1	31.7	2.64	0	3	1	0	0	0	2	0	1	82	1	0	
96	1	16.3	1.36	1	3	1	0	0	0	2	0	1	80	1	1	0
97	1	18.2	1.52	1	3	1	0	0	0	2	0	1	44	0	0	1
98	1	64	5.33	0	3	1	0	0	0	2	0	0	39	0	0	0
99	1	39.1	3.26	1	3	1	0	0	0	2	0	1	71	1	1	1
100	1	69.2	5.77	0	3	1	0	0	0	2	0	1	58	0	0	0
101	1	33.4	2.78	1	3	1	0	0	0	2	0	1	60	1	0	0
102	1	9.23	0.77	1	3	1	0	0	0	2	0	1	80	1	0	1
103	1	76.6	6.38	0	3	1	0	0	0	2	0	1	78	1	0	0
104	1	15.2	1.27	1	3	1	0	0	0	2	0	0	59	0	0	0
105	1	3.4	0.28	1	3	1	0	0	0	2	0	1	59	0	0	1
106	1	62.5	5.21	0	3	1	0	0	0	2	0	1	73	1	0	0
107	1	31.6	2.63	1	3	1	0	0	0	2	0	1	78	1	0	0
108	1	66.1	5.51	0	3	1	0	0	0	2	0	0	69	1	0	0
109	1	65.9	5.49	0	3	1	0	0	0	2	0	0	58	0	0	0
110	1	67.9	5.66	0	3	1	0	0	0	2	0	1	65	1	0	0
111	1	72	6.00	0	3	1	0	0	0	2	0	1	56	0	0	
112	1	62.7	5.23	0	3	1	0	0	0	2	0	0	77	1	1	0
113	1	62.5	5.21	0	3	1	0	0	0	2	0	0	60	1	0	0
114	1	63	5.25	0	3	1	0	0	0	2	0	1	17	0	0	0
115	1	67.9	5.66	0	3	1	0	0	0	2	0	0	78	1	0	0
116	1	45.3	3.78	1	3	1	0	0	0	2	0	1	64	1	0	0
117	1	19.13	1.59	1	3	1	0	0	0	2	0	1	60	1	1	0
118	1	43.23	3.60	1	3	1	0	0	0	2	0	1	80	1	0	0
119	1	42.7	3.56	1	3	1	0	0	0	2	0	0	64	1	0	0
120	1	66.1	5.51	0	3	1	0	0	0	2	0	0	48	0	0	1
121	1	0.5	0.04	1	3	1	0	0	0	2	0	1	73	1	0	0
122	1	85.4	7.12	1	3	1	0	0	0	2	0	1	71	1	0	0
123	1	18.96	1.58	1	3	1	0	0	0	2	0	1	73	1	0	0
124	1	69.6	5.80	0	3	1	0	0	0	2	0	1	41	0	0	1
125	1	48.83	4.07	1	3	1	0	0	0	2	0	0	52	0	0	0
126	1	62.4	5.20	0	3	1	0	0	0	2	0	1	65	1	0	0
127	1	19.06	1.59	1	4	1	0	0	0	2	0	0	69	1	1	
128	1	19.06	1.59	1	4	1	0	0	0	2	0	0	69	1	1	
129	1	18.4	1.53	1	4	1	0	0	1	4	1	0	71	1	1	0
130	1	66.9	5.58	0	2	0	1	1	0	3	1	0	92	1	0	0
131	1	67.9	5.66	0	2	0	1	1	0	3	1	1	48	0	1	0
132	1	28.66	2.39	1	3	1	1	1	0	3	1	0	78	1	0	0
133	1	37.1	3.09	1	3	1	1	1	0	3	1	1	82	1	0	0
134	1	71	5.92	0	3	1	1	1	0	3	1	0	67	1	0	0
135	1	70.7	5.89	0	3	1	1	1	0	3	1	1	64	1	1	0
136	1	83	6.92	1	3	1	1	1	0	3	1	1	70	1	0	0
137	1	58.4	4.87	0	3	1	1	1	0	3	1	1	49	0	0	0
138	1	1.76	0.15	1	3	1	1	1	0	3	1	0	70	1	0	1
139	1	6.43	0.54	1	3	1	1	1	0	3	1	1	68	1	0	1
140	1	69.7	5.81	0	3	1	1	1	0	3	1	0	73	1	0	
141	1	66.4	5.53	0	3	1	1	1	0	3	1	1	68	1	0	0
142	1	34.4	2.87	1	3	1	1	1	0	3	1	1	52	0	0	0
143	1	66.5	5.54	0	3	1	1	1	0	3	1	1	43	0	0	0
144	1	67.5	5.63	0	3	1	1	1	0	3	1	1	62	1	0	0
145	1	68.3	5.69	0	3	1	1	1	0	3	1	1	78	1	0	0
146	1	68.5	5.71	0	3	1	1	1	0	3	1	1	71	1	0	0
147	1	66.5	5.54	0	3	1	1	1	0	3	1	1	60	1	1	0
148	1	79.6	6.63	0	3	1	1	1	0	3	1	0	58	0	0	0
149	1	28.1	2.34	1	3	1	1	1	0	3	1	0	72	1	0	0
150	1	64.7	5.39	0	3	1	1	1	0	3	1	0	68	1	0	0

151	1	32	2.67	1	3	1	1	1	0	3	1	1	70	1	1	1
152	1	30.06	2.51	1	2	0	2	1	0	3	1	1	56	0	1	0
153	1	12.43	1.04	1	3	1	2	1	0	3	1	1	79	1	1	1
154	1	67.5	5.63	0	3	1	2	1	0	3	1	0	59	0	0	0
155	1	15	1.25	1	3	1	2	1	0	3	1	1	70	1	0	1
156	1	19.63	1.64	1	3	1	2	1	1	4	1	1	58	0	1	1
157	1	19	1.58	1	3	1	0	0	1	4	1	0	82	1	0	0
158	1	18.4	1.53	1	3	1	1	1	1	4	1	1	71	1	0	0
159	1	7.36	0.61	1	3	1	1	1	1	4	1	1	51	0	1	0
160	1	7.36	0.61	1	3	1	1	1	1	4	1	1	51	0	1	0
161	1	35	2.92	1	3	1	1	1	1	4	1	1	38	0	0	

Table S2. Primary antibodies used in this study.

Source	Primary antibodies	Catalog no.	Working dilution
Abmart	Anti-NXPH4 antibody produced in rabbit	TP73307	WB: 1:1000 IHC: 1:500 CoIP: 5μg
ProteinTech	Anti-pan-keratin antibody produced in rabbit	26411-1-AP	mIHC: 1:1000
Novusbio	Anti-NSUN4 antibody produced in rabbit	NBP2-19594	WB: 1:1000 RIP: 5μg
Novusbio	Anti-PHD4 antibody produced in rabbit	NB100-295	WB: 1:1000 CoIP: 5μg
ProteinTech	Anti-HIF1A antibody produced in rabbit	20960-1-AP	WB: 1:1000 CoIP: 5μg ChIP: 5μg
ProteinTech	Anti-GFP antibody produced in rabbit	50430-2-AP	RIP: 5μg
Diagenode	Anti-m^5C antibody produced in mouse	C15200003	RIP: 5μg
ABclonal	Anti-ACTB antibody produced in rabbit	AC026	WB: 1:10000

Table S3. Primers used for qPCR.

Target gene	Primer sequence (5'-3') Forward	Reverse	Size (bp)
NXPH4	CAAGCGTGTCGAGTTCGGA	GAAAGCGCGTGACTCTTTGG	220
18s rRNA	CAGCCACCCGAGATTGAGCA	TAGTAGCGACGGGCGGTGTG	252
GAPDH	AACGGATTTGGTCGTATTGGG	CCTGGAAGATGGTGATGGGAT	211
ZIC5	GTCTATGGGCCTGATTGTGTAGT	GTCTATGGGCCTGATTGTGTAGT	83
FOXD1	TGAGCACTGAGATGTCCGATG	CACCACGTCGATGTCTGTTTC	59
TIMP1	AGAGTGTCTGCGGATACTTCC	CCAACAGTGTAGGTCTTGGTG	169
HES4	GAGCGCGTATTAACGAGAGCCT	CTCACGGTCATCTCCAGGATGT	118
CCNF	CACAAAGCATCCATATTGCACTG	TGGTCAGACATCCCTGATGAG	118

Table S4. Primers used for ChIP assay.

Target promoter	Primer sequence (5'-3') Forward	Reverse	Size (bp)
ZIC5	TTTTGAAACTGGGACTGCCC	GATTCGCCCCCGTGCTTG	243
FOXD1	CCGAGAAGGGCTGATTTCCT	GGGCGGGCGAATCAGAAC	203
TIMP1	GGCGGCTTGGAAGGAATAGT	TAAATGTCCACGCTAGGGGC	233
HES4	CAAGGACGTCGCTCACGA	CGCTGCCTGGTAACTCGG	152
CCNF	CACAGGGCGGGACATGG	AAGGGGTGTGGGGCAGAAA	248

Table S5. Guide RNA used for targeted RNA methylation system.

Target transcript	gRNA sequence (5'-3')
NXPH4 gRNA1	AGAACACTGGGAAGGGGGTCCCA
NXPH4 gRNA2	CTTTCACTTTGGGGTGTACTGGA
NXPH4 gRNA3	GAGTTATTAAAGCTGCGGGGAAA

Table S6. shRNA used for silencing target genes.

Target transcript	Sequence (5'-3')
NXPH4 sh1#	CCTAAGACTGTAAAGGCCTAA
NXPH4 sh2#	CATCTGTATCTTCGTCTCTTT
shYTHDF1	CCCTACCTGTCCAGCTATTAC
shDNMT1	GCCCAATGAGACTGACATCAA
shNOP2	GACGATGCTGATACGGTAGAT
shALYREF	CGTGGAGACAGGTGGGAAACT
shWTAP	ATGGCAAGAGATGAGTTAATT
shHNRNPC	GCGCTTGTCTAAGATCAAATT
NSUN4 sh1#	GATGGAAATCAAGTTCGAGTT
NSUN4 sh2#	GCTGGTAATACCAAACCTCAT
OPTN sh1#	CGTGATGGATTGCATCATTTA
OPTN sh2#	GCCAAGAATTACTTCGAACAT
NBR1 sh1#	CCATCCTACAATATCTGTGAA
NBR1 sh2#	GCAGTTAAACAGGGAAACCAA
TAX1BP1 sh1#	GCACAACATGAAAGAGAACAA
TAX1BP1 sh2#	GCTACTTTGAGTTTGGTGTTA
CALCOCO2 sh1#	CCTGACTTGATACTAAGTGAT
CALCOCO2 sh2#	GAGCTGCTTCAACTGAAAGAA
LAMP2C sh1#	GCCATCAGAATTCCATTGAAT
LAMP2C sh2#	GAAGTGAACATCAGCATGTAT
SQSTM1 sh1#	CCTCTGGGCATTGAAGTTGAT
SQSTM1 sh2#	CCGAATCTACATTAAAGAGAA

NXPH4 antibody

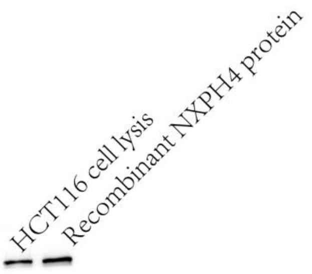

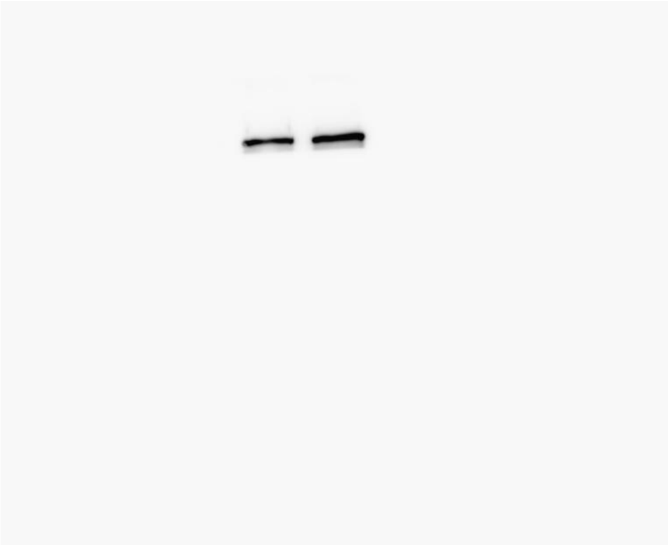

Figure2A

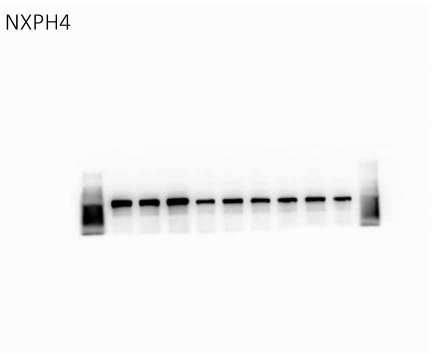

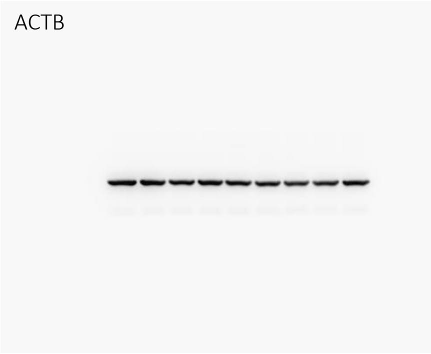

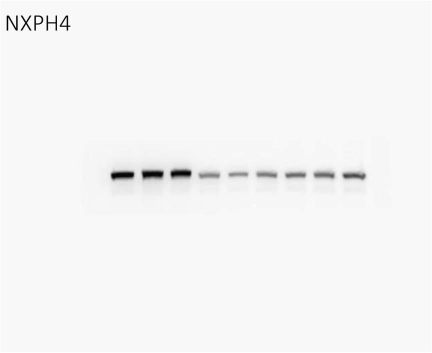

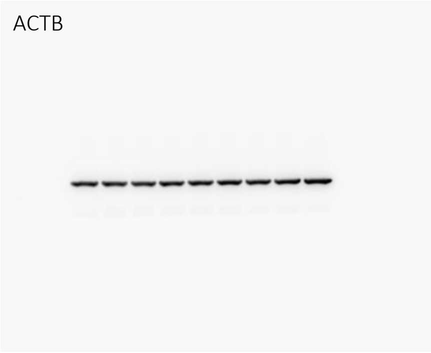

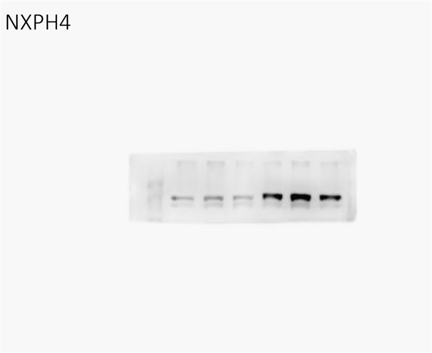

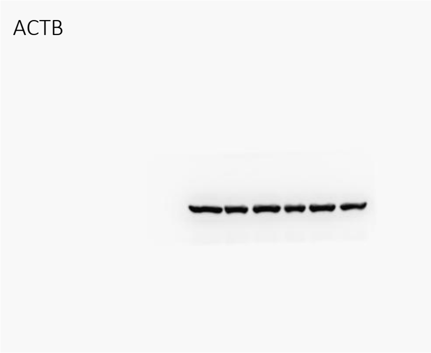

NXPH4 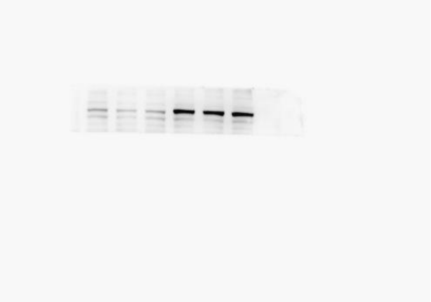	ACTB

Figure3A

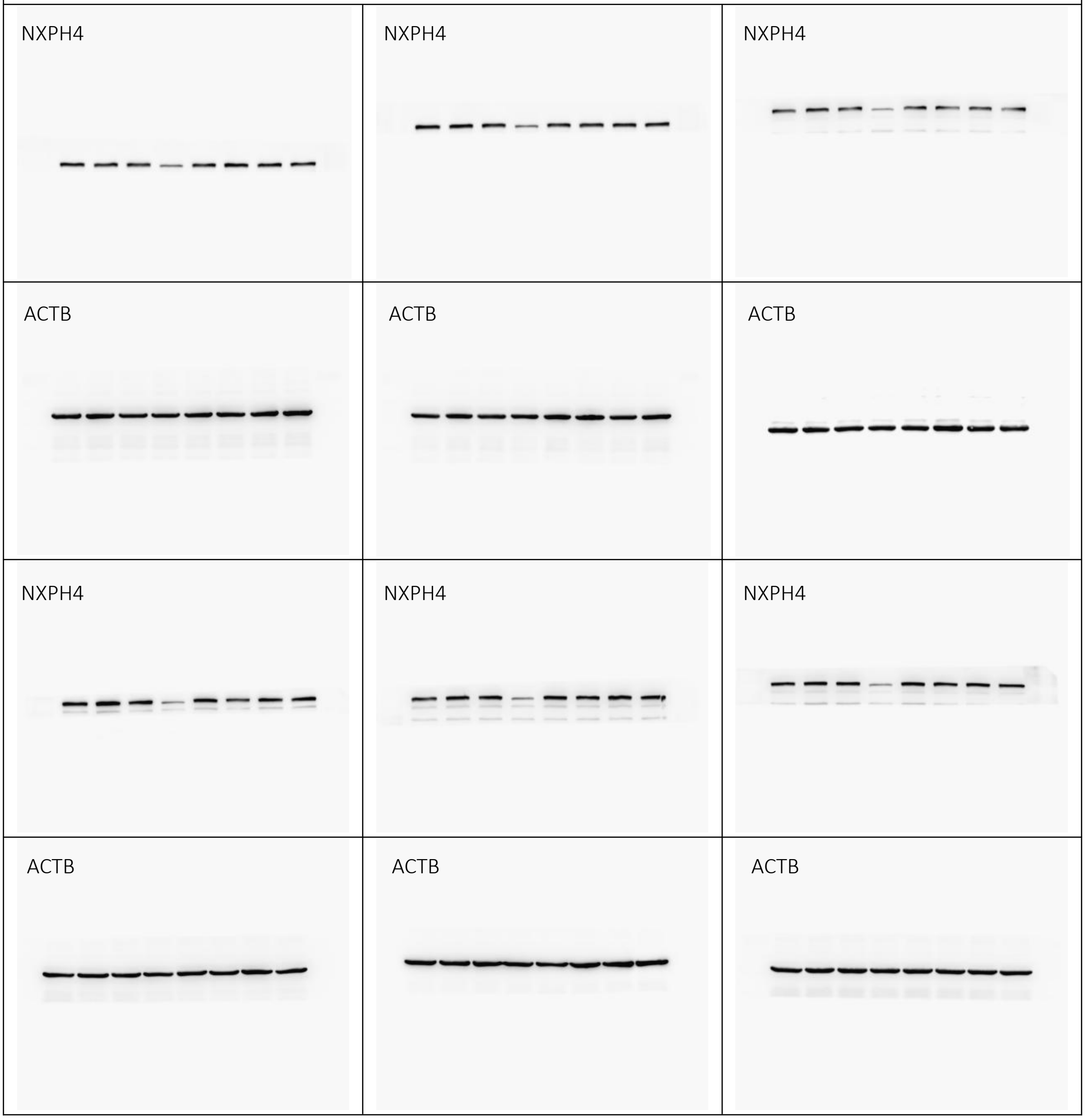

Figure3D

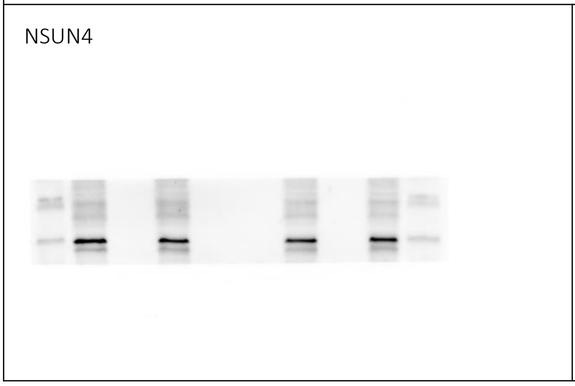

Figure4E

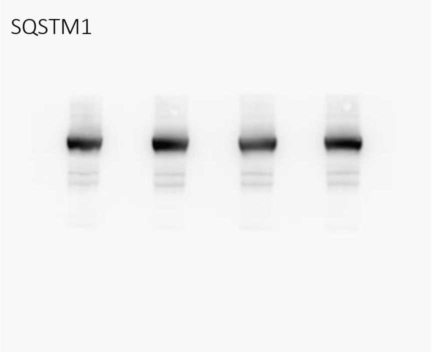

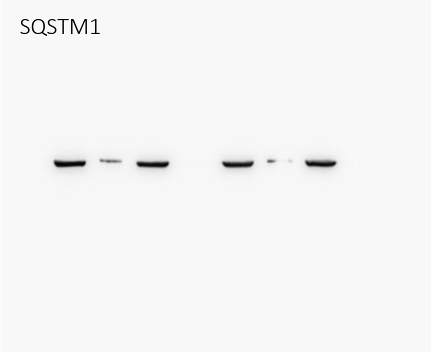

Figure4F

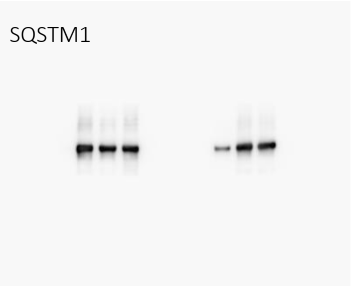

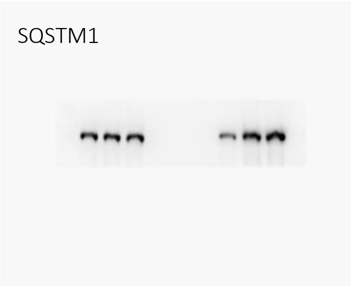

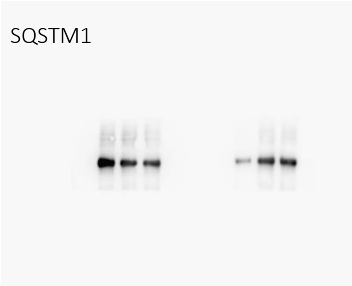

Figure4G

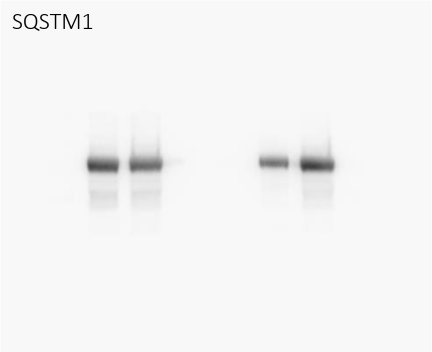

Figure4H

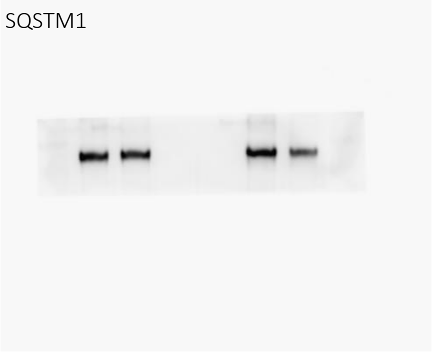

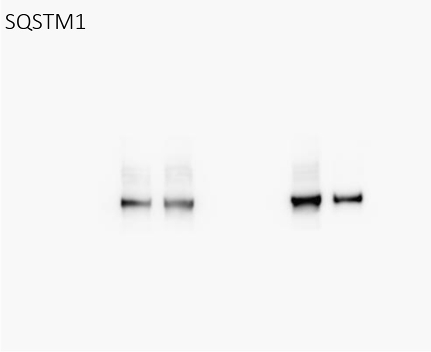

Figure5E

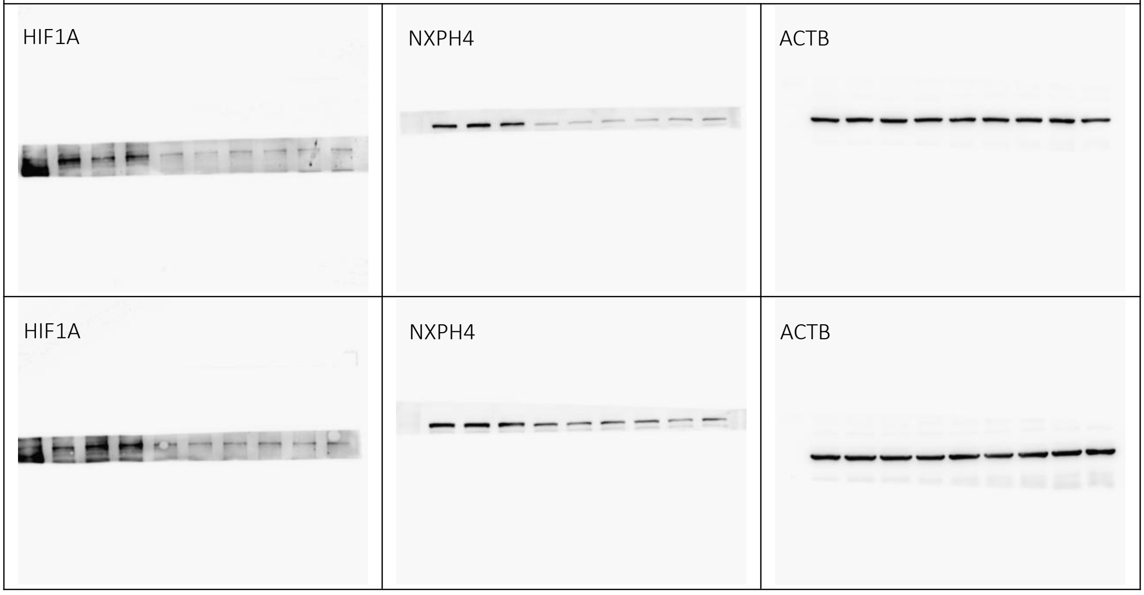

Figure5H

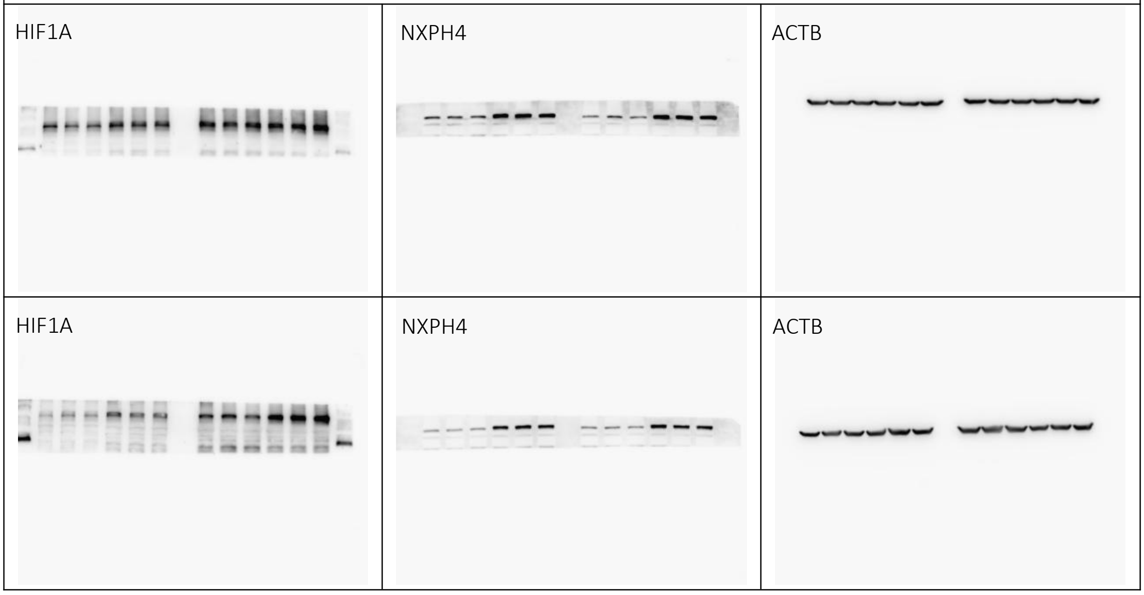

Figure6A

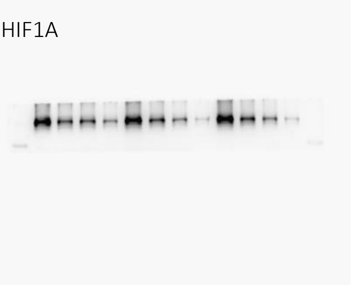
HIF1A

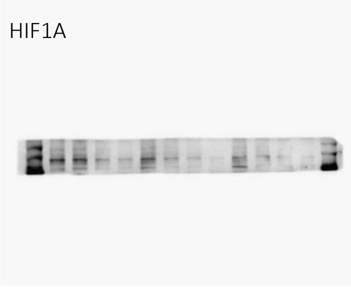
HIF1A

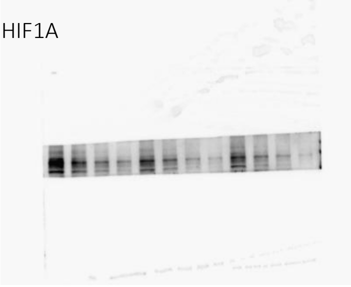
HIF1A

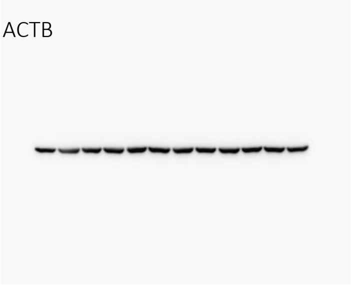
ACTB

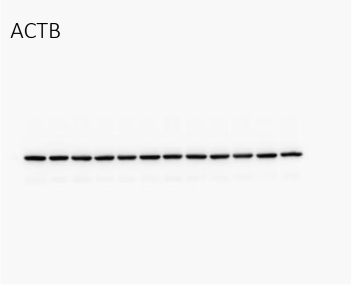
ACTB

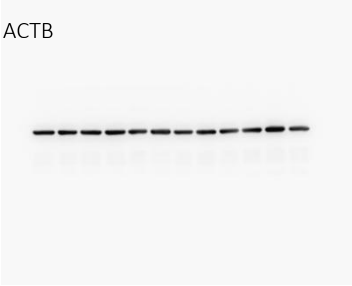
ACTB

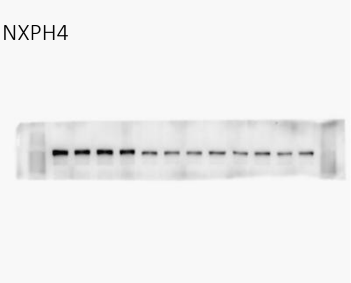
NXPH4

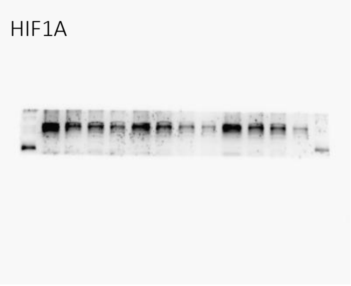
HIF1A

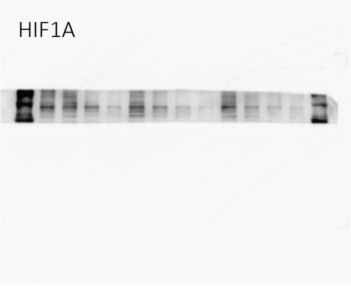
HIF1A

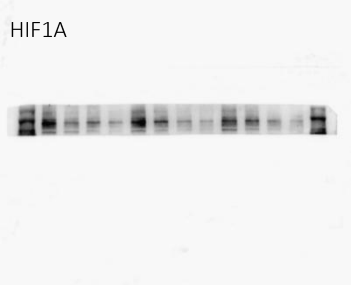
HIF1A

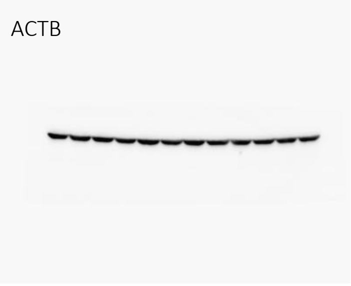
ACTB

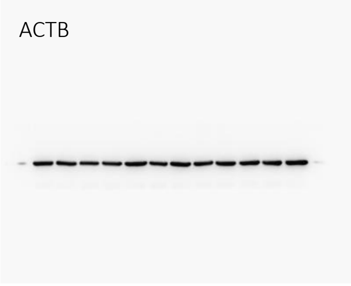
ACTB

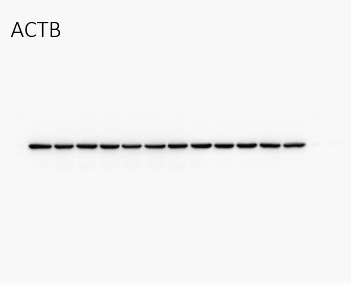
ACTB

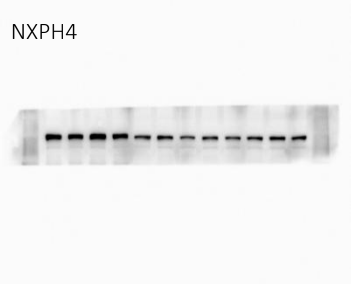
NXPH4

Figure6D

HIF1A 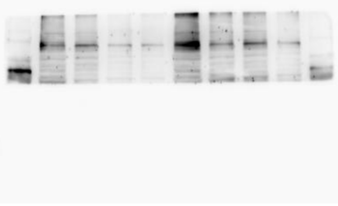	HIF1A 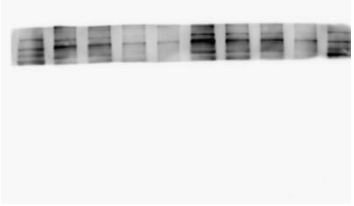	HIF1A 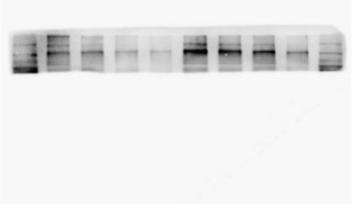
ACTB 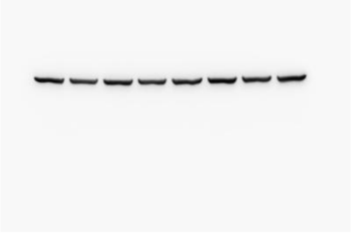	ACTB	ACTB
NXPH4		
HIF1A 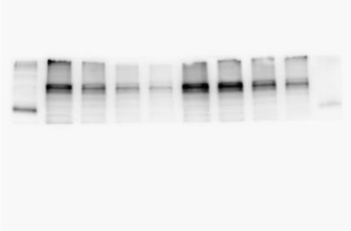	HIF1A 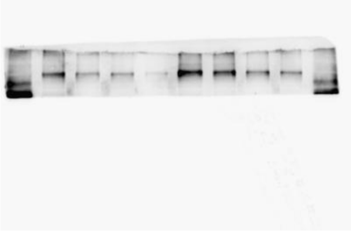	HIF1A 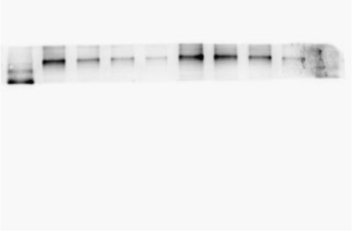
ACTB	ACTB	ACTB
NXPH4 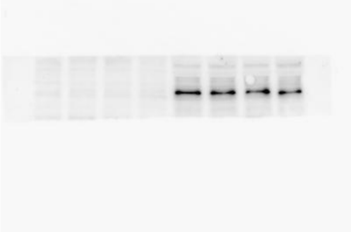		

Figure6H & S19A

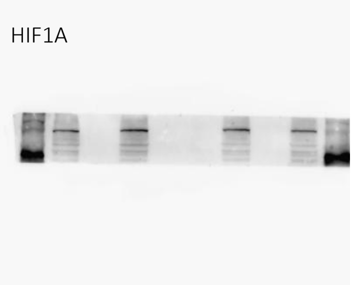

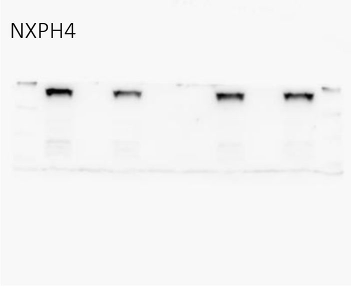

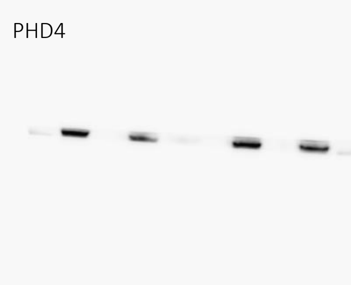

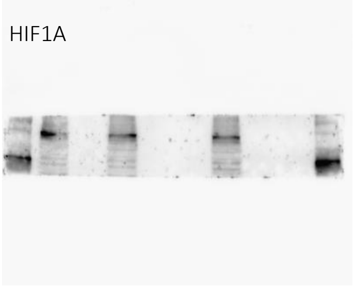

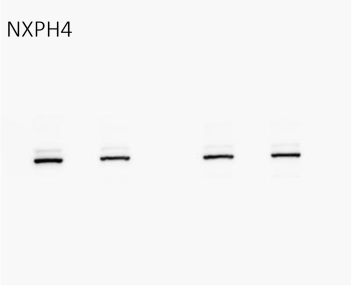

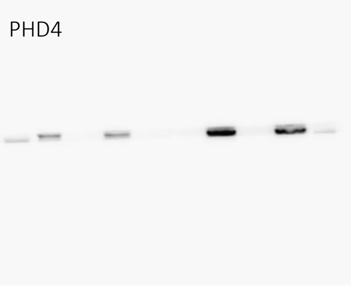

Figure6I

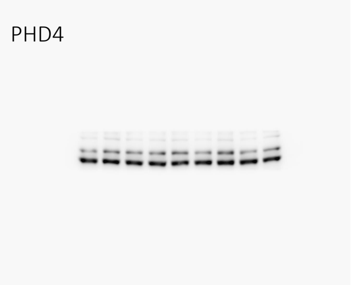

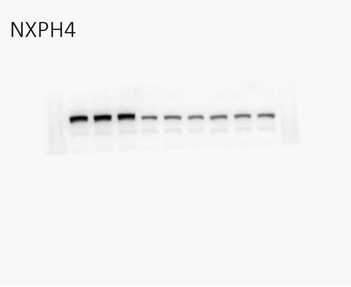

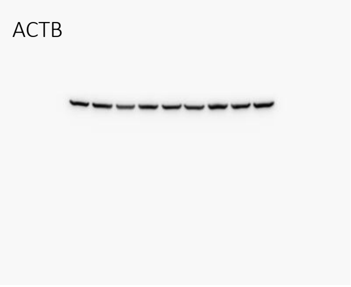

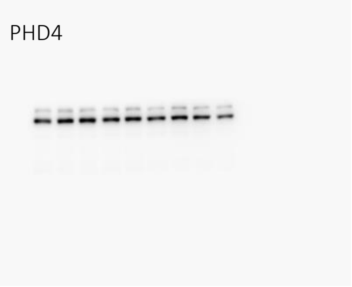

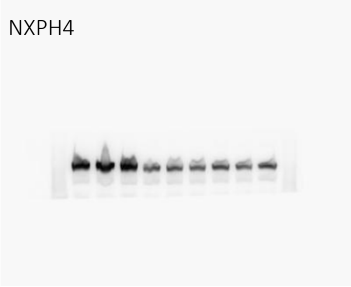

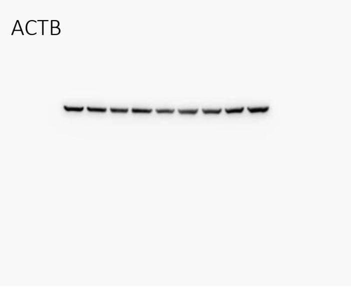

Figure6J

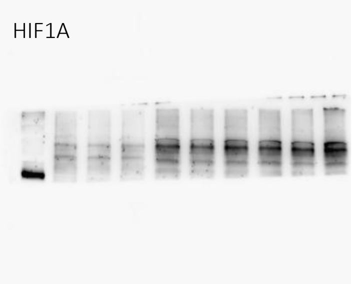

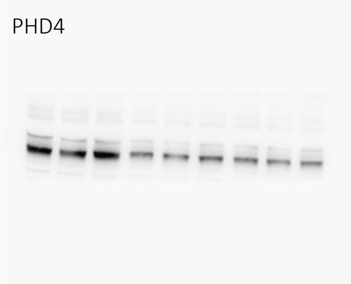

ACTB

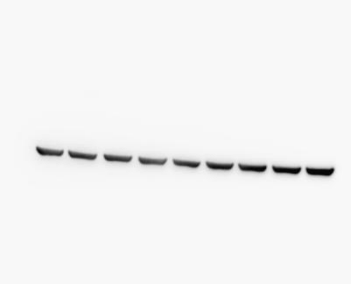

Figure6L		
HIF1A 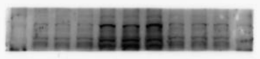	PHD4 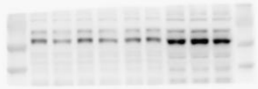	
NXPH4 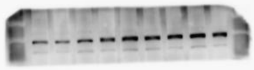	ACTB	

Figure6O		
HIF1A 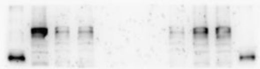	HIF1A 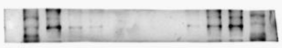	HIF1A 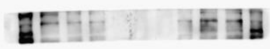
PHD4 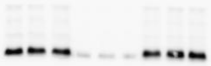	PHD4	PHD4 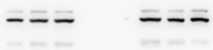

Figure6Q & S20C		
HIF1A 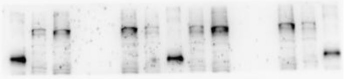	HIF1A 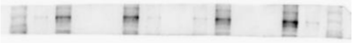	HIF1A 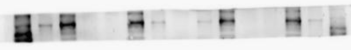

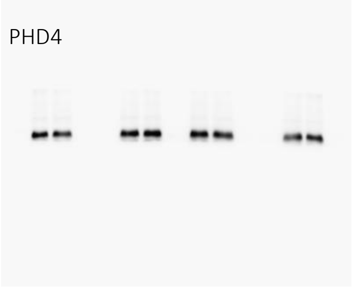

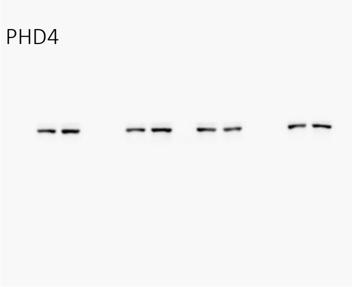

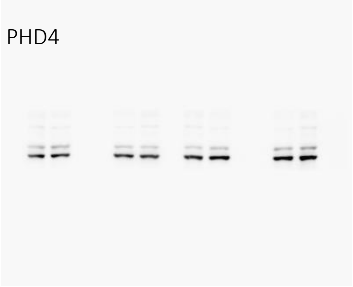

Figure S3A

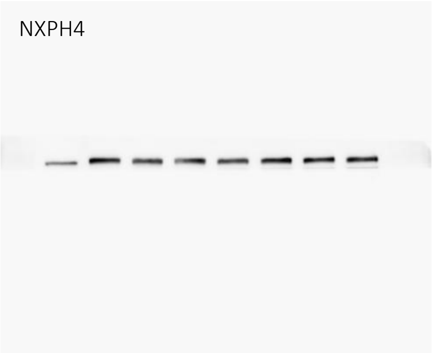

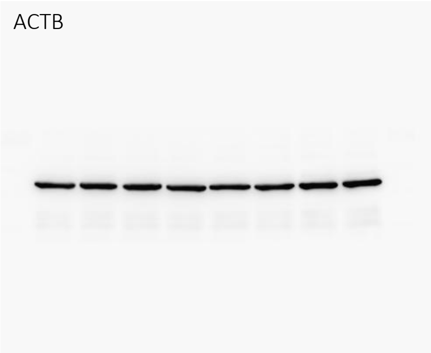

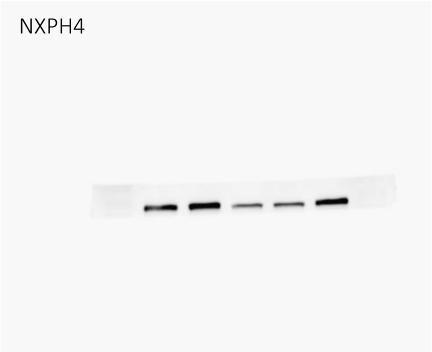

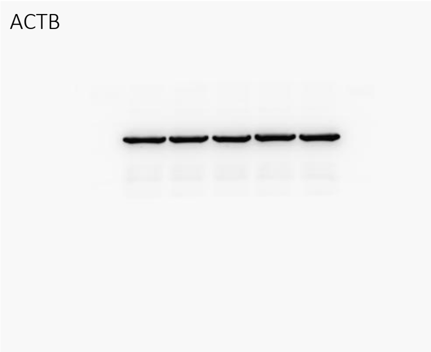

Figure S3E

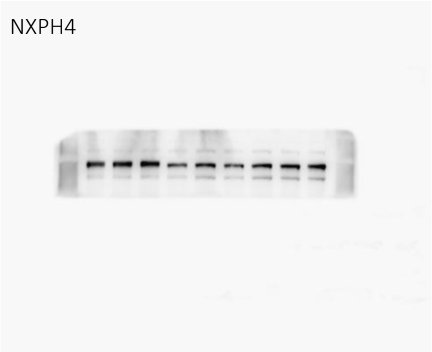

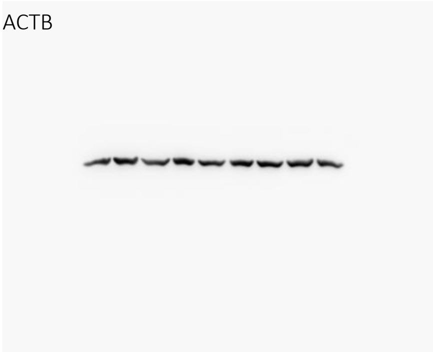

NXPH4 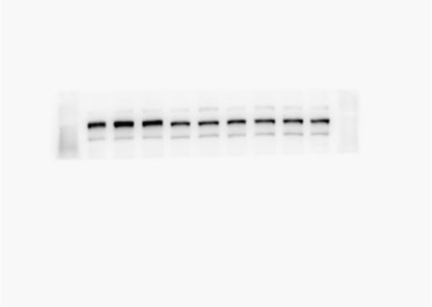	ACTB 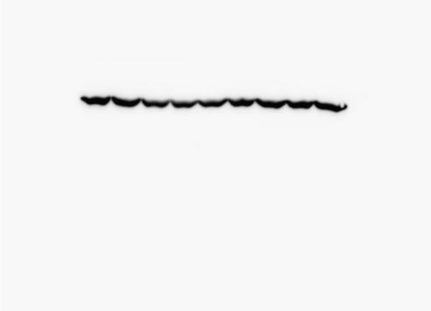
NXPH4	ACTB
NXPH4	ACTB

FigureS8A

NXPH4 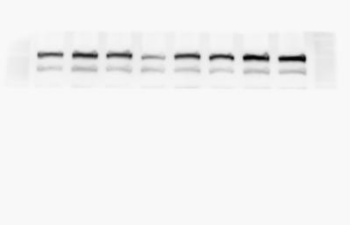	NXPH4 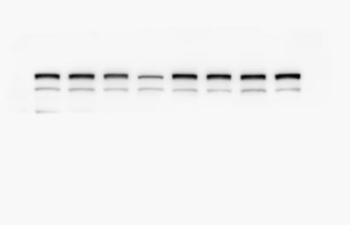	NXPH4 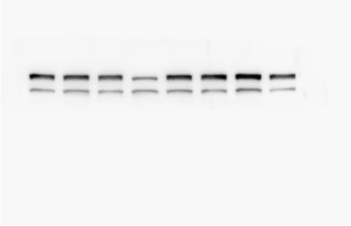

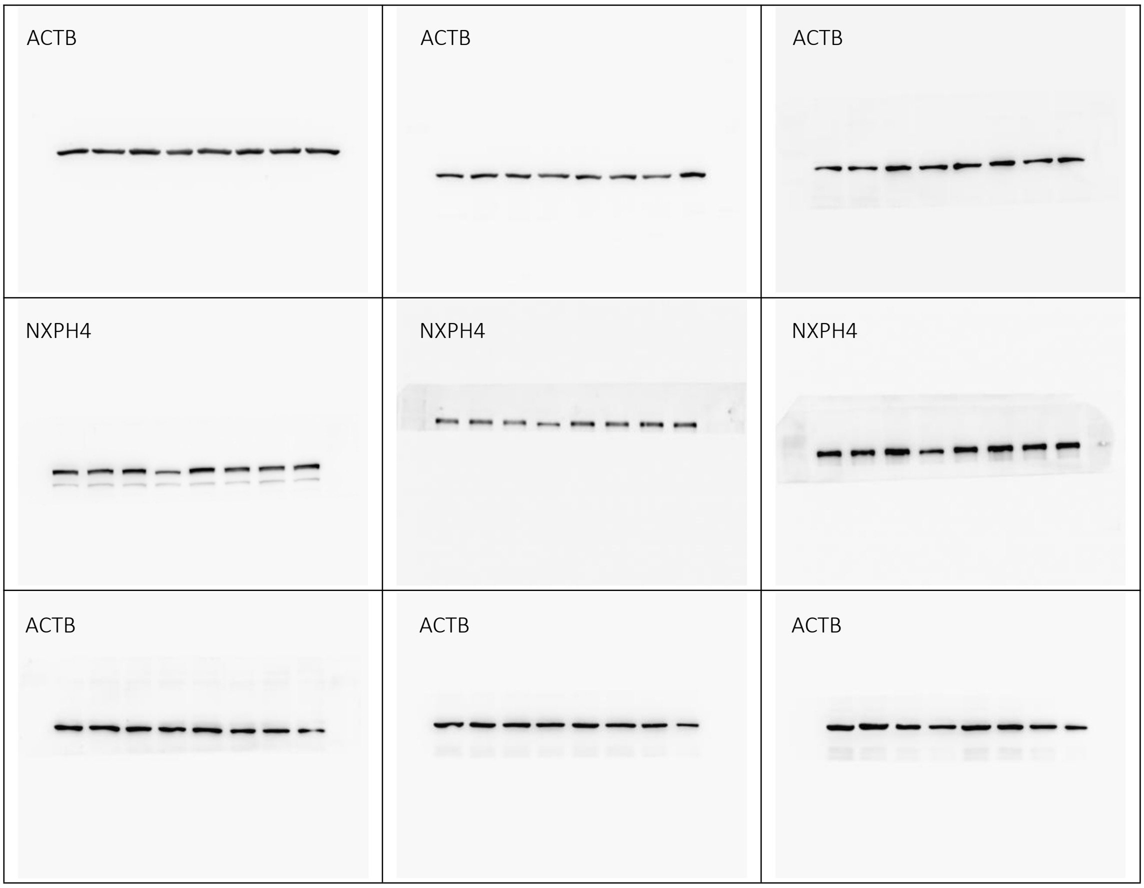
ACTB
ACTB
ACTB
NXPH4
NXPH4
NXPH4
ACTB
ACTB
ACTB

Figure S15A

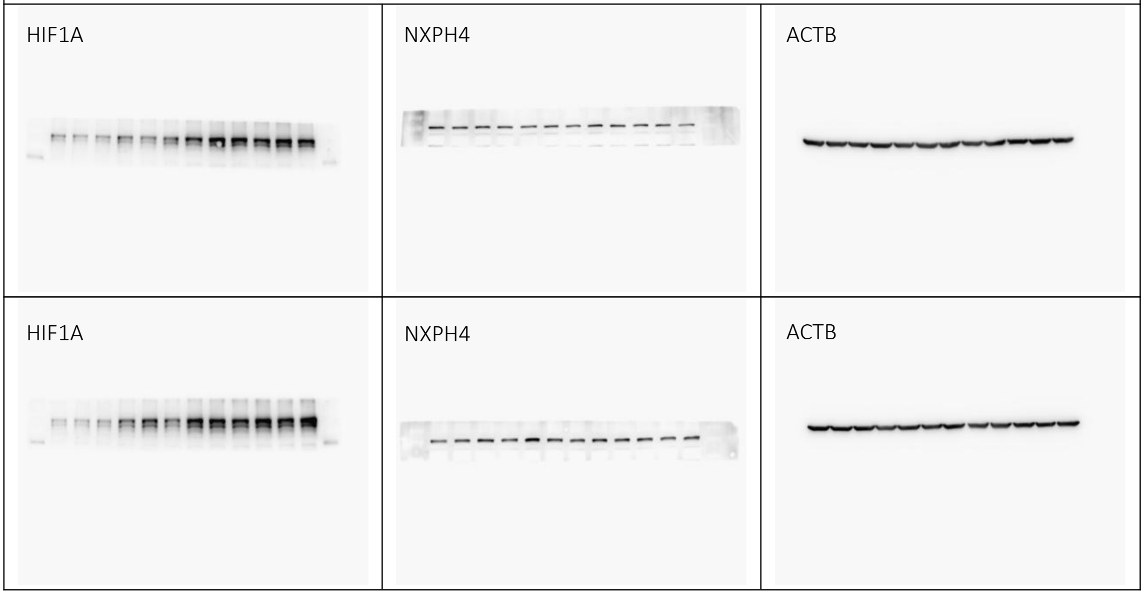
HIF1A
NXPH4
ACTB
HIF1A
NXPH4
ACTB

Figure S15D

HIF1A

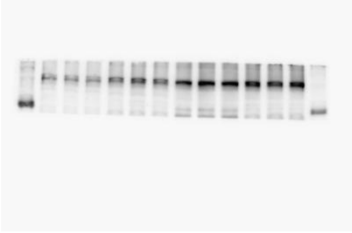

NXPH4

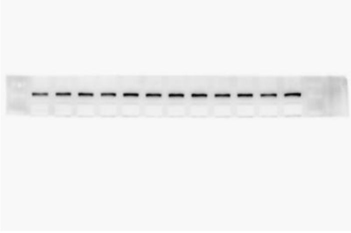

ACTB

HIF1A

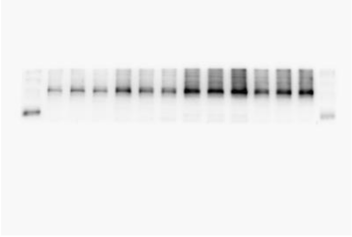

NXPH4

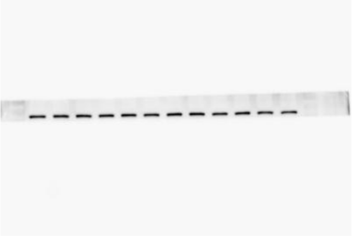

ACTB

Figure S15G

HIF1A

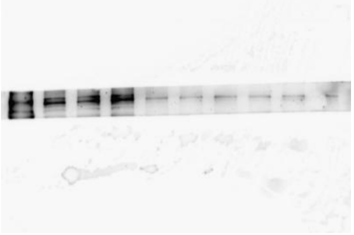

NXPH4

ACTB

HIF1A

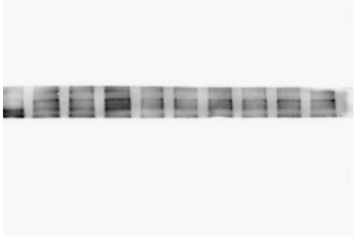

NXPH4

ACTB

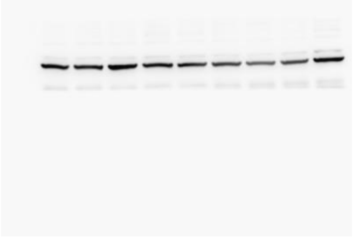

Figure S15L

HIF1A

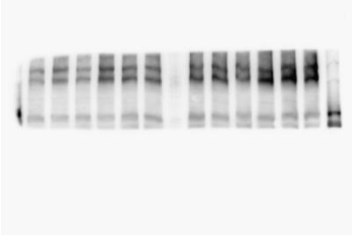

NXPH4

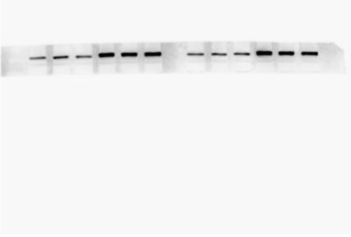

ACTB

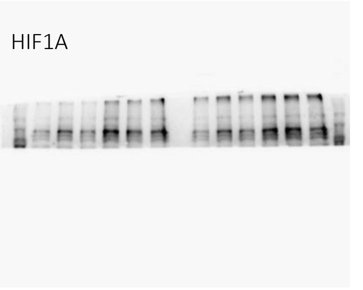

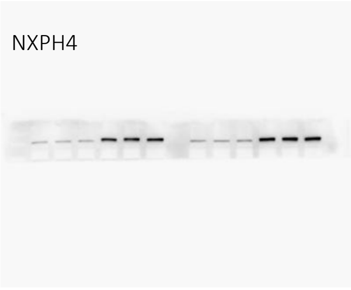

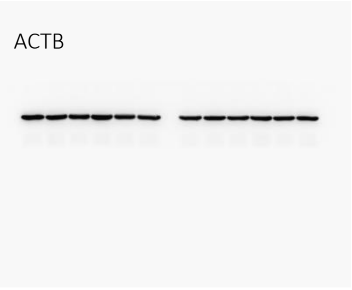

Figure S15N

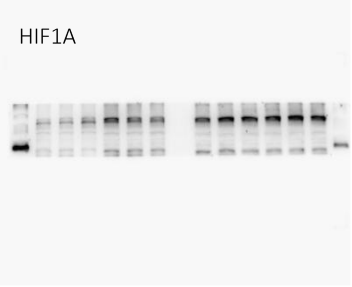

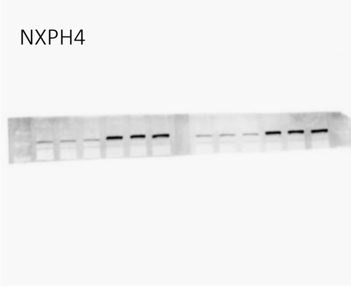

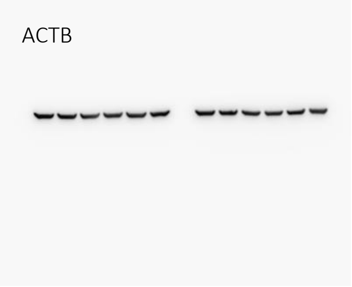

Figure S18A

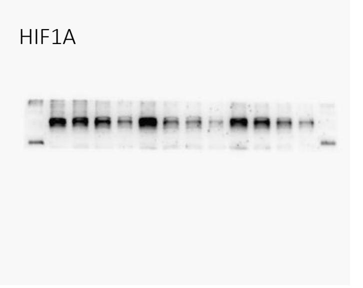

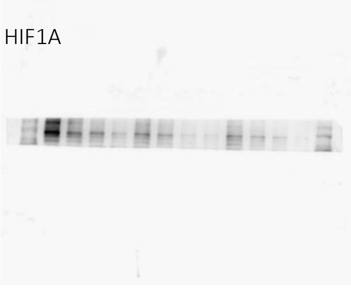

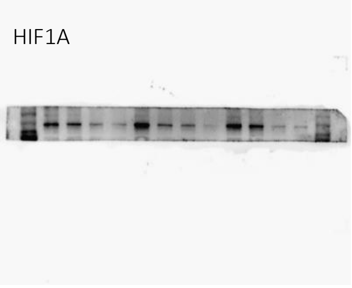

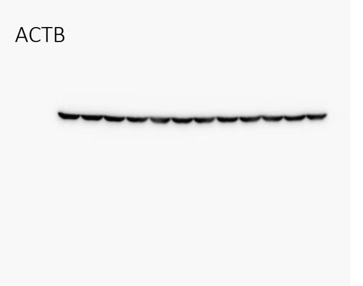

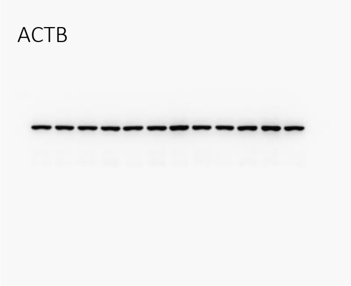

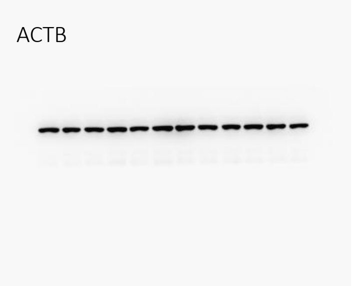

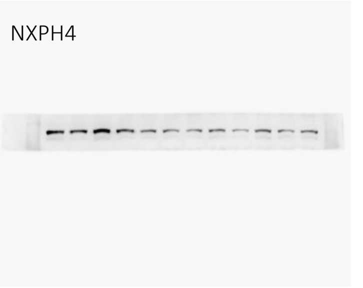

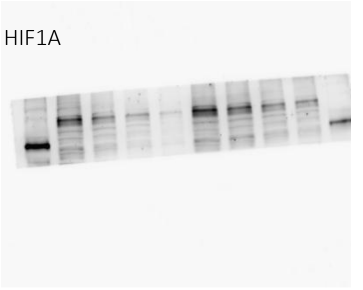

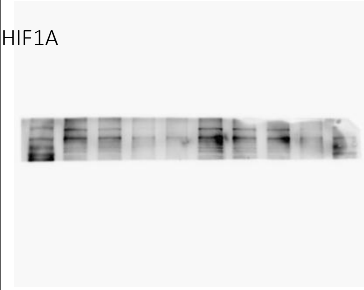

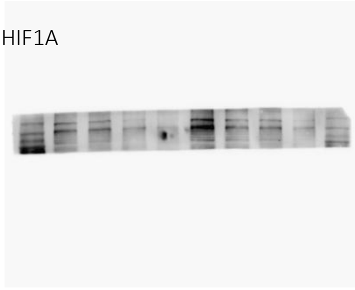

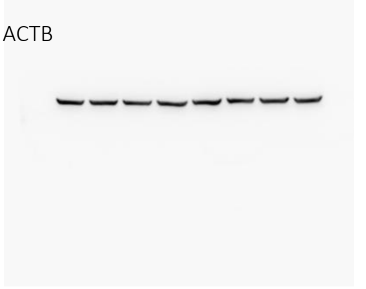

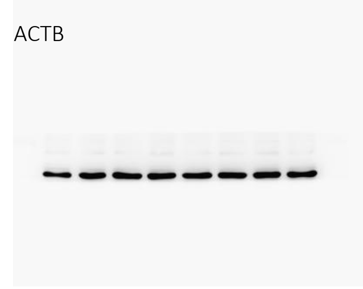

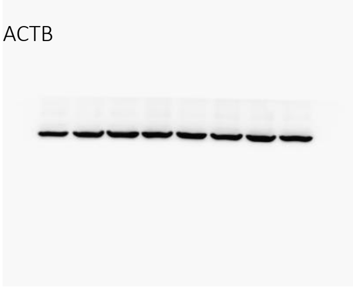

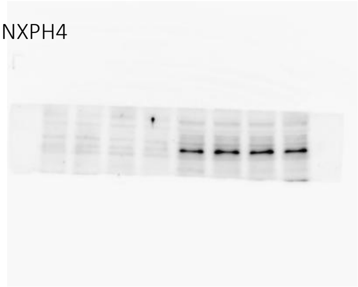

Figure S19B

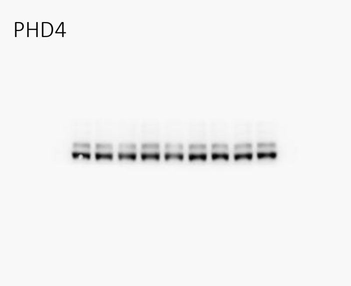

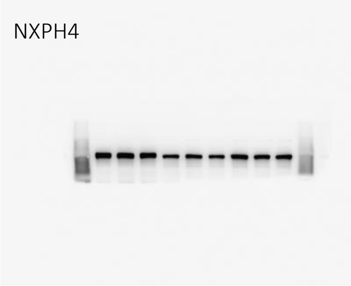

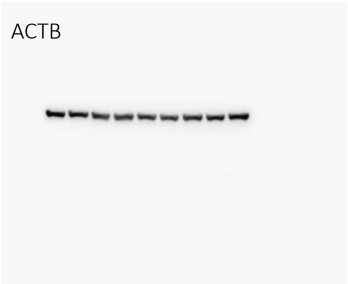

Figure S19D

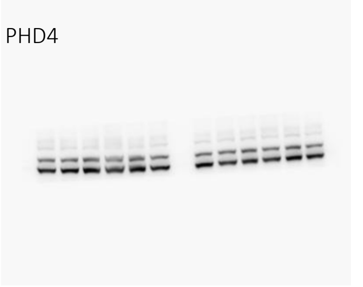

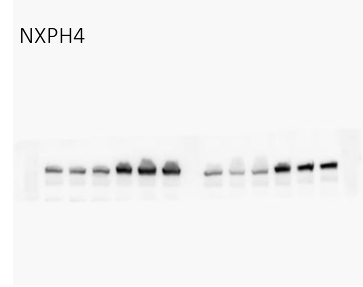

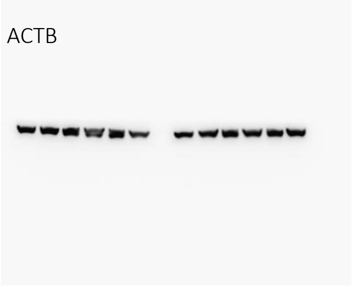

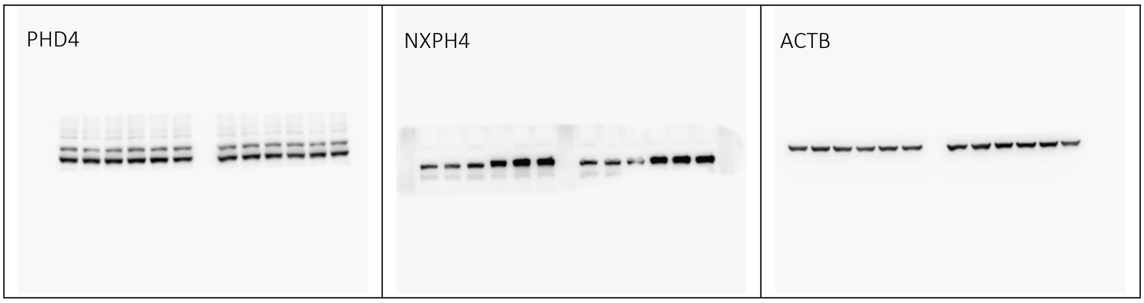
PHD4
NXPH4
ACTB

Figure S19G

HIF1A

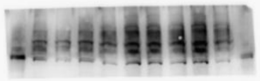

PHD4

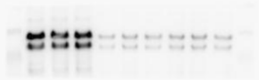

ACTB

HIF1A

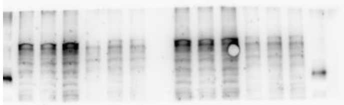

PHD4

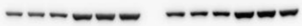

ACTB

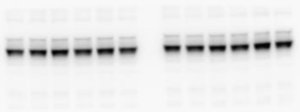

Figure S19K

HIF1A

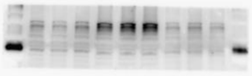

PHD4

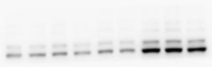

NXPH4

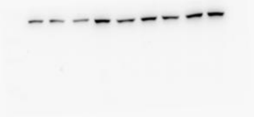

ACTB

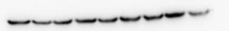

HIF1A

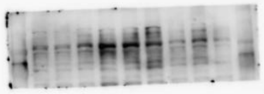

PHD4

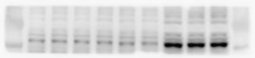

NXPH4 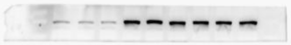	ACTB	

FigureS19O		
HIF1A 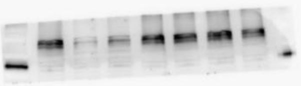	HIF1A 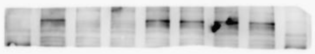	HIF1A 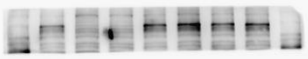
ACTB	ACTB 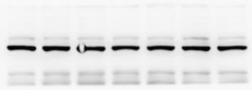	ACTB 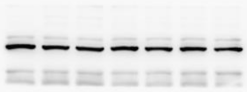
PHD4 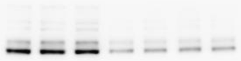		
NXPH4 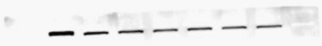		

HIF1A 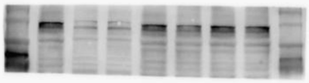	HIF1A 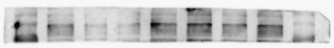	HIF1A 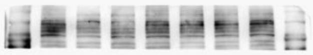
ACTB	ACTB	ACTB
PHD4 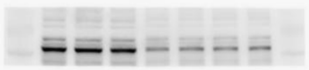		
NXPH4		
HIF1A 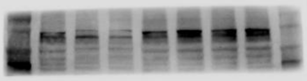	HIF1A 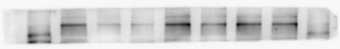	HIF1A 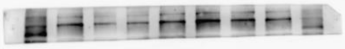
ACTB	ACTB	ACTB

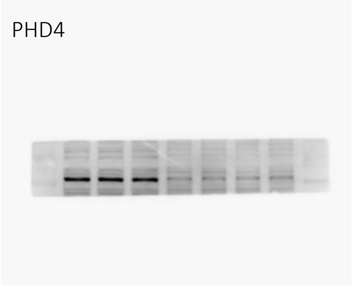 PHD4		
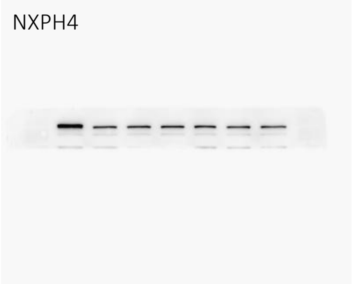 NXPH4		

Figure S20A

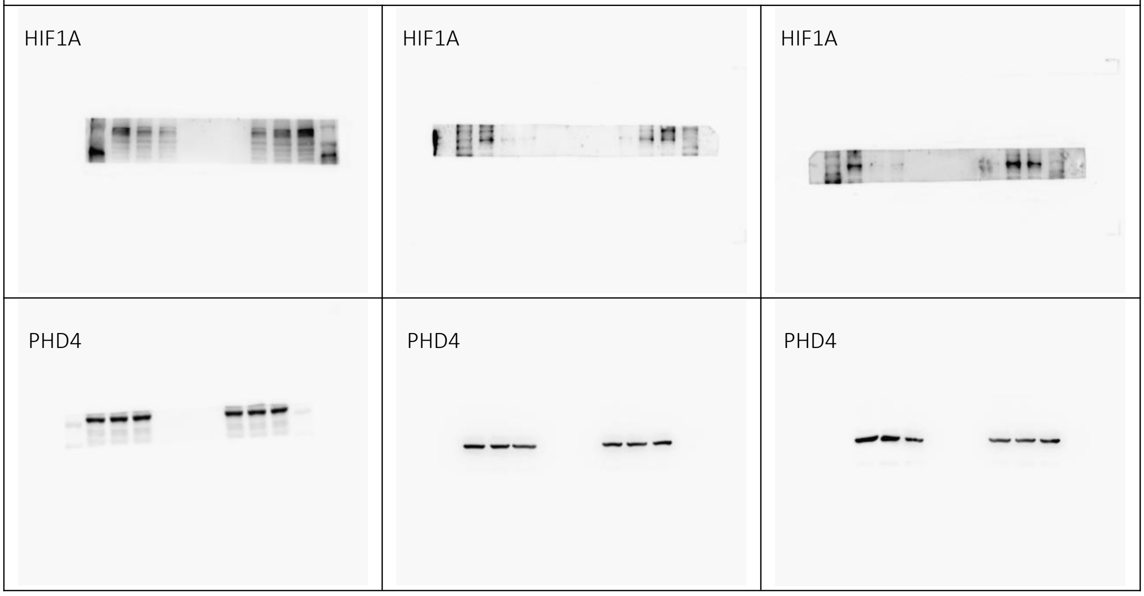

Supplement: Supplementary file 1 — Supplementary Material 1. [file 11658_2024_630_MOESM1_ESM.pdf]
